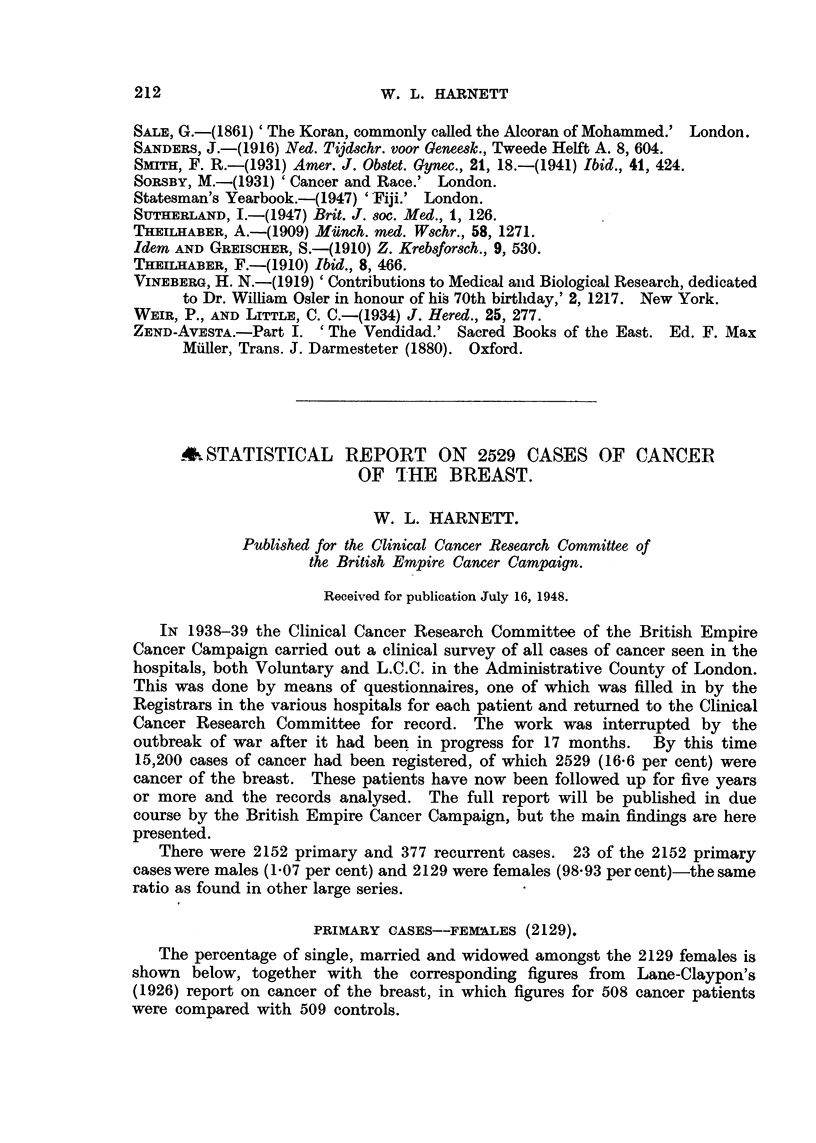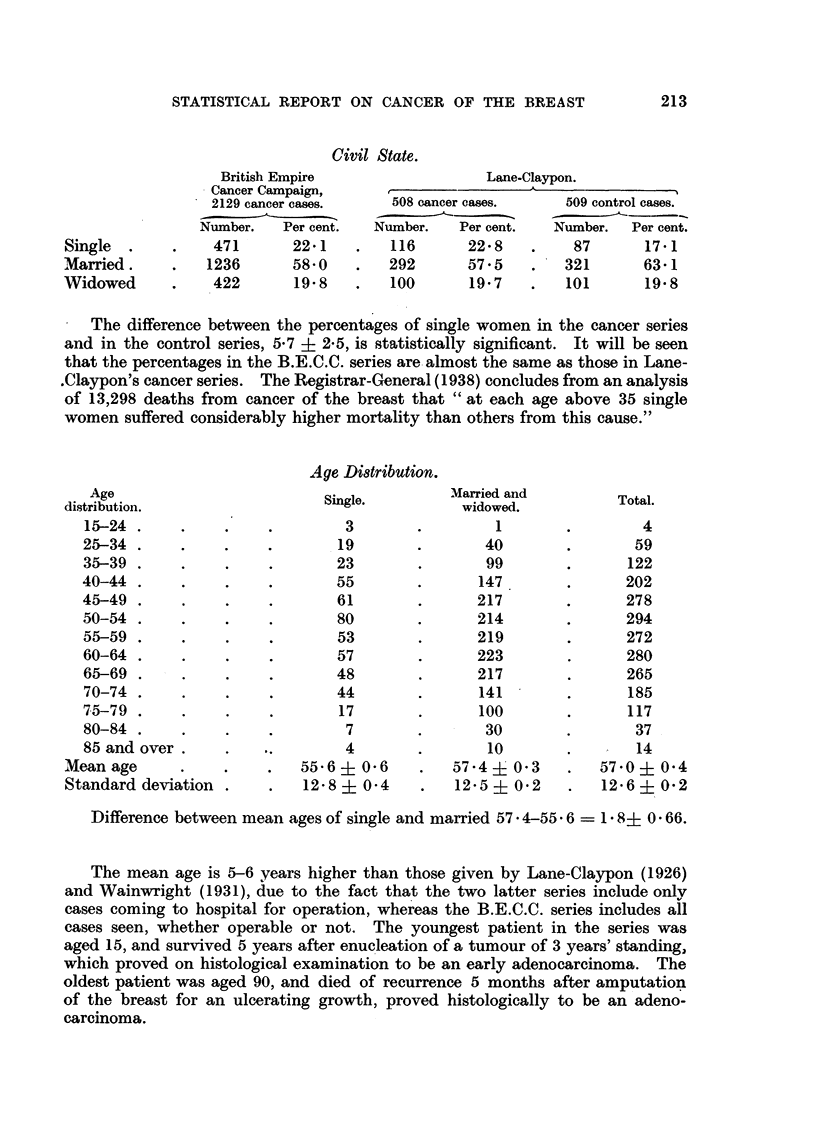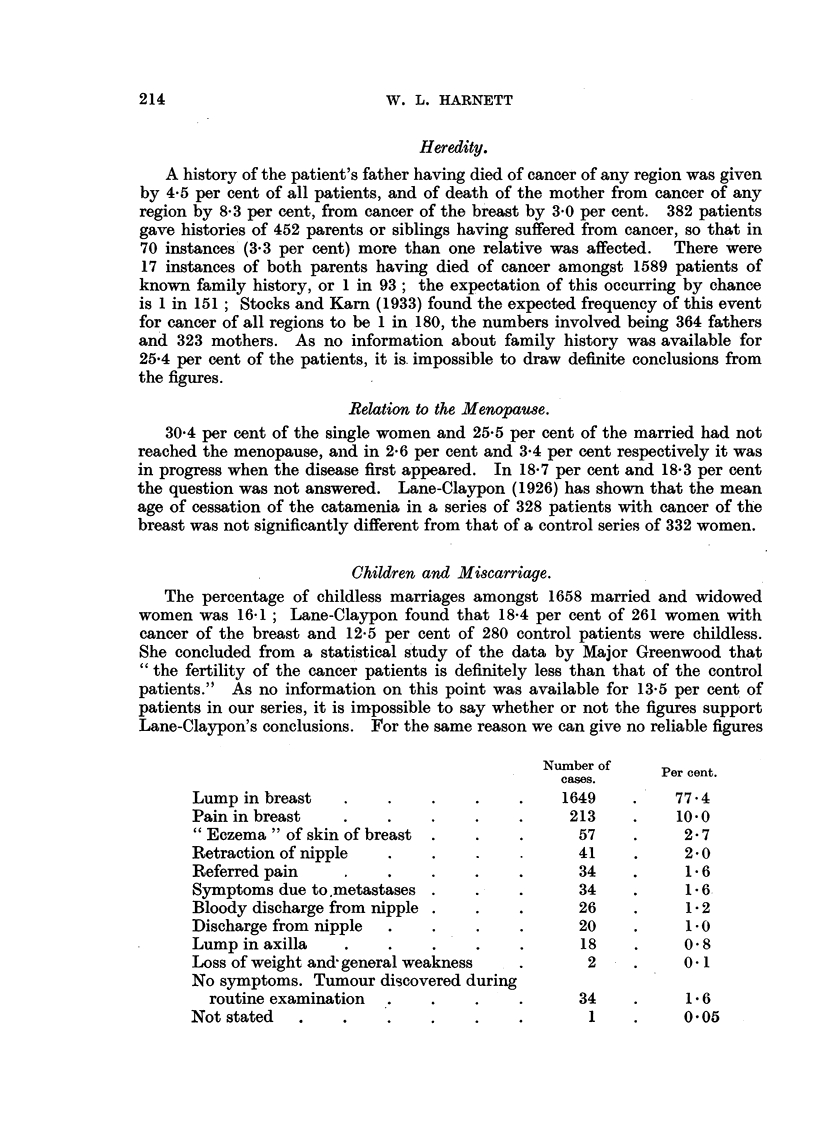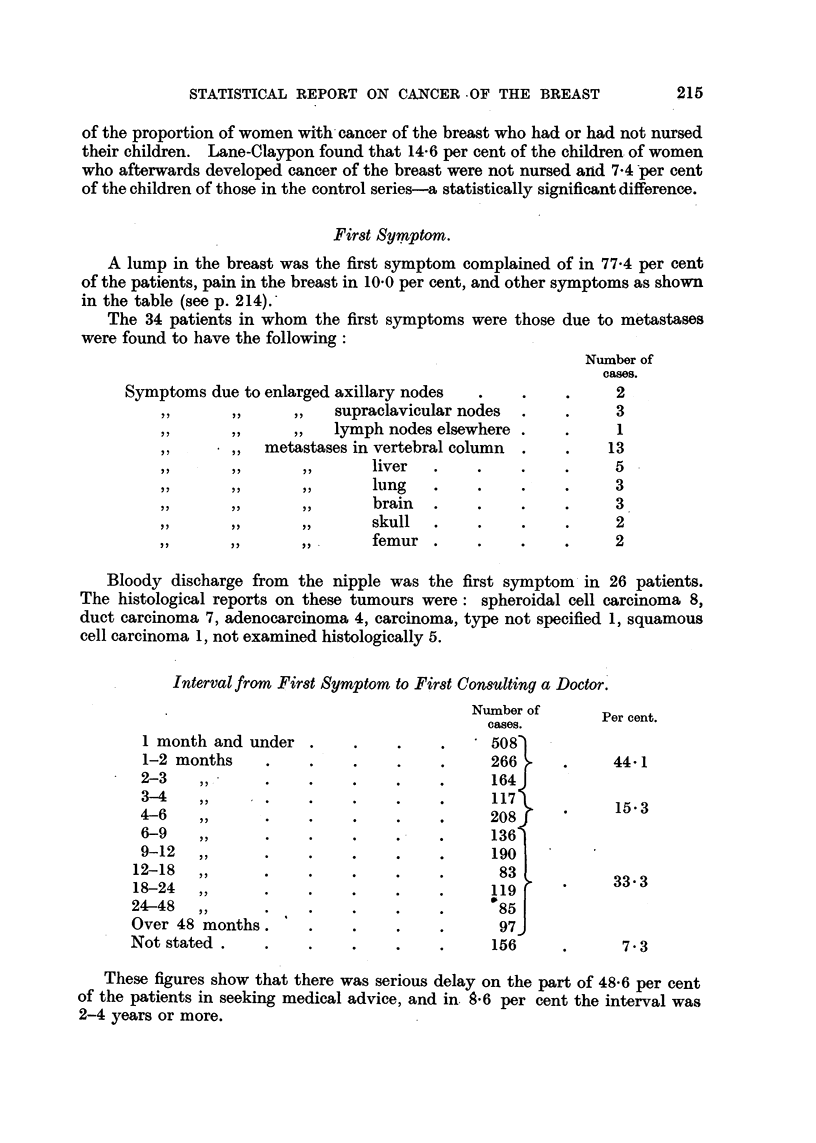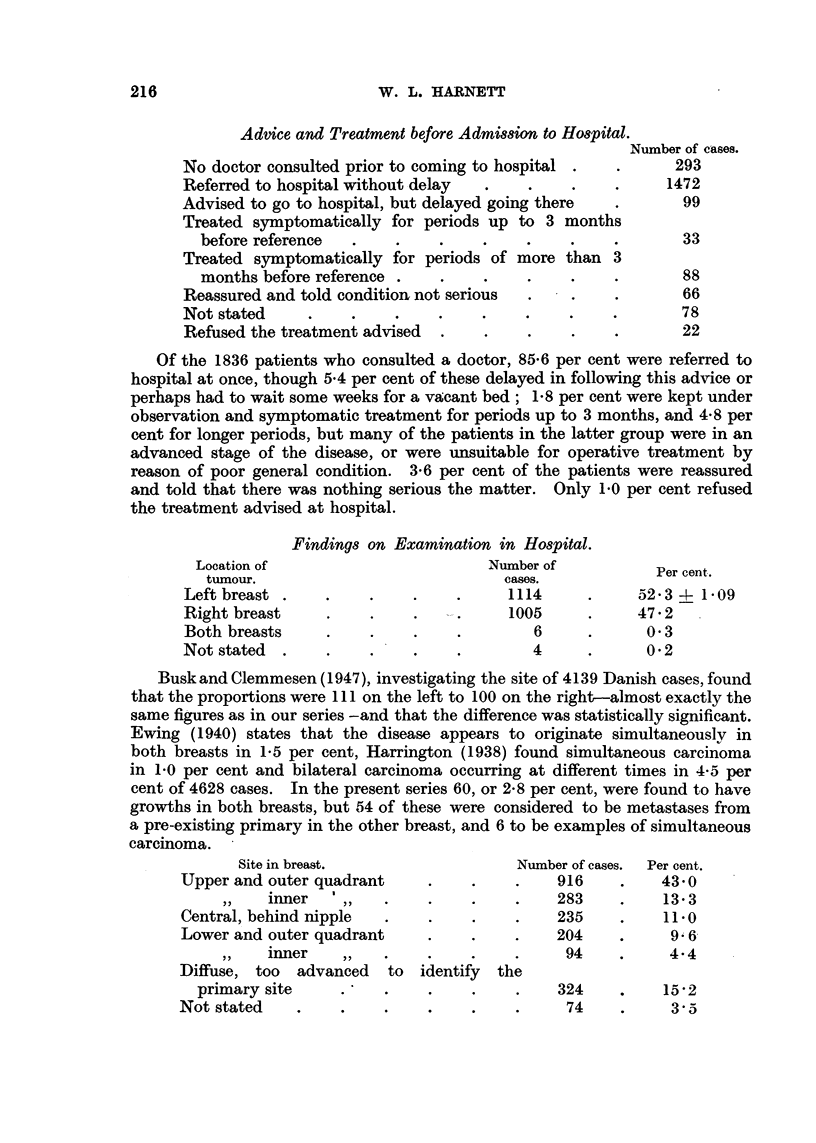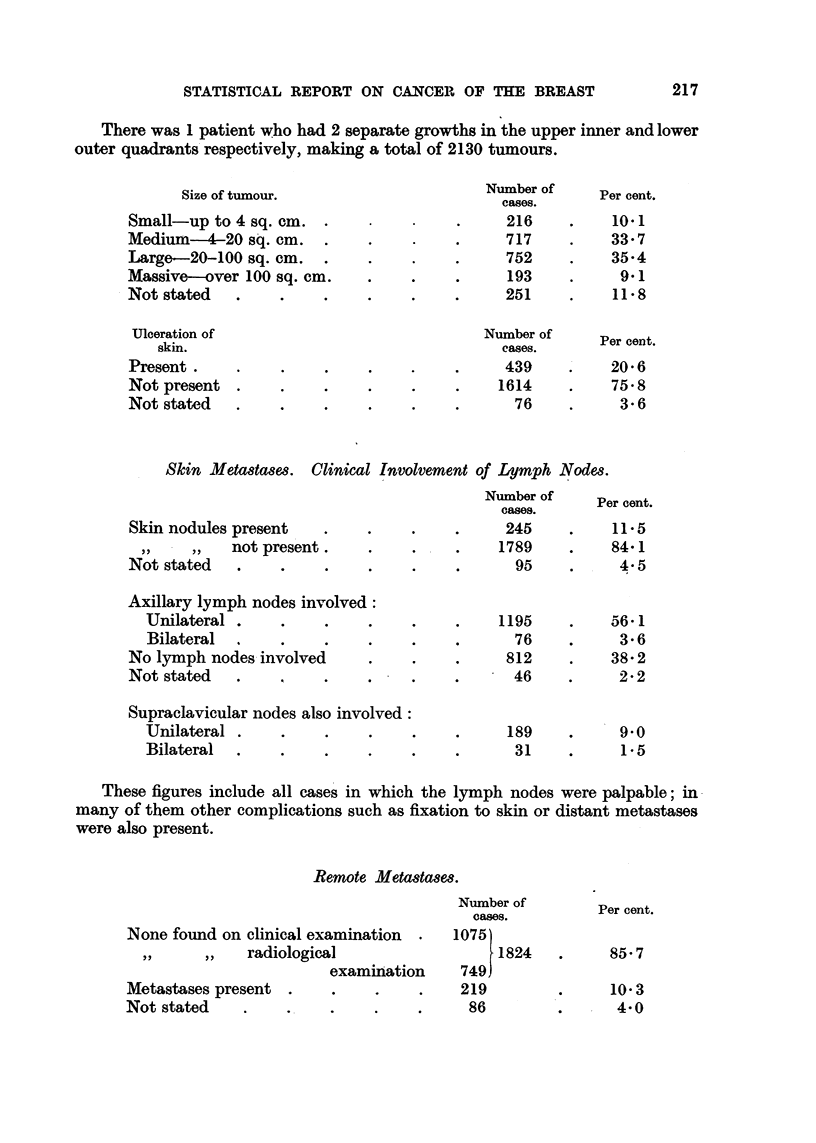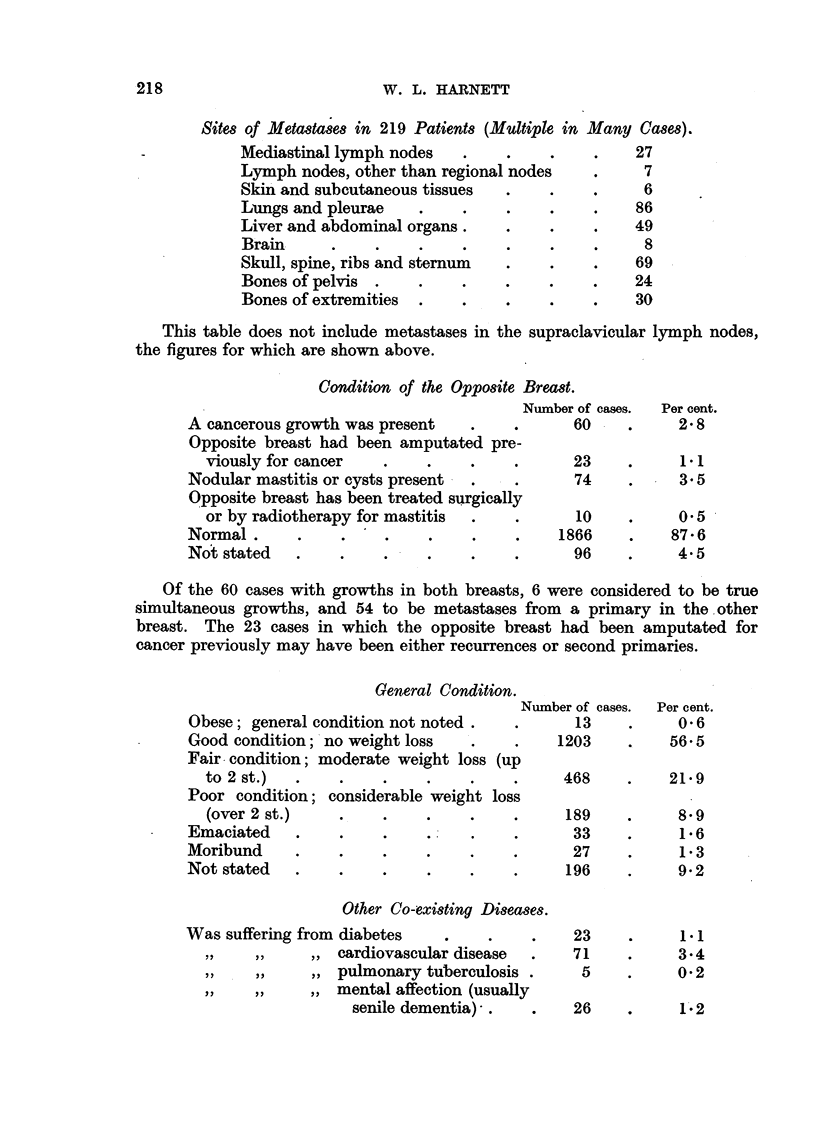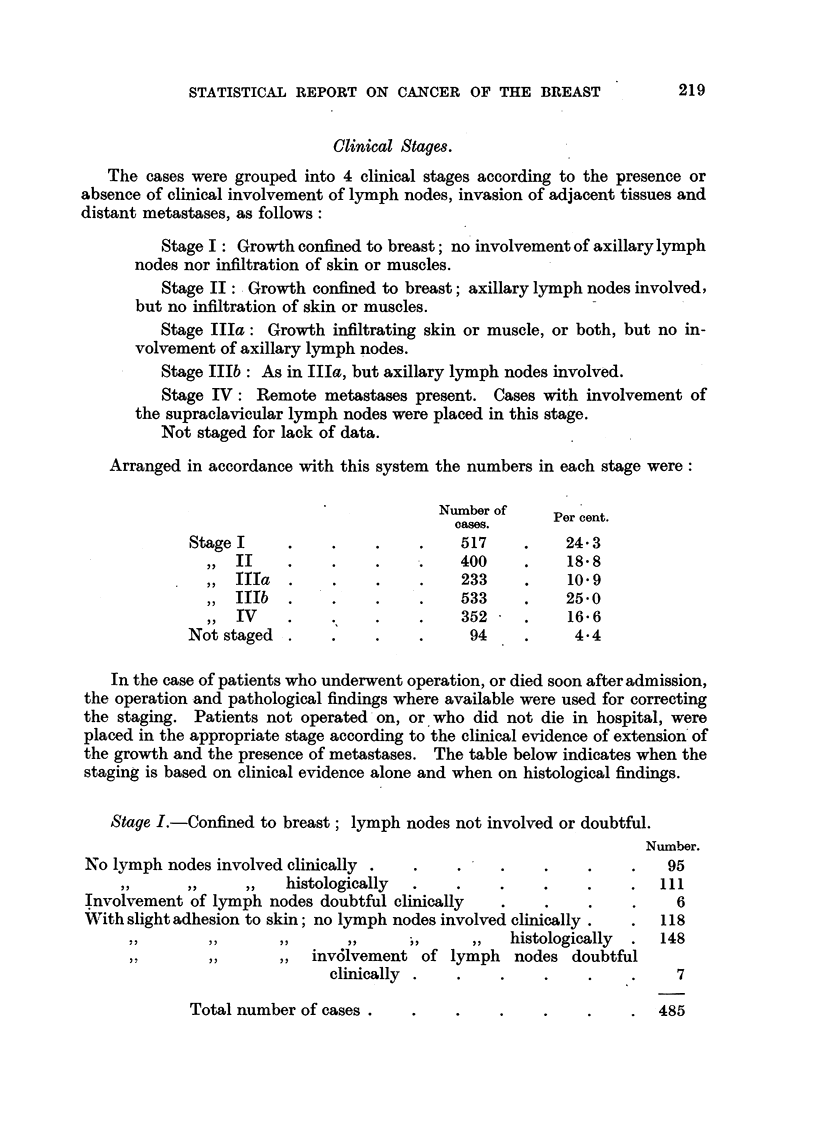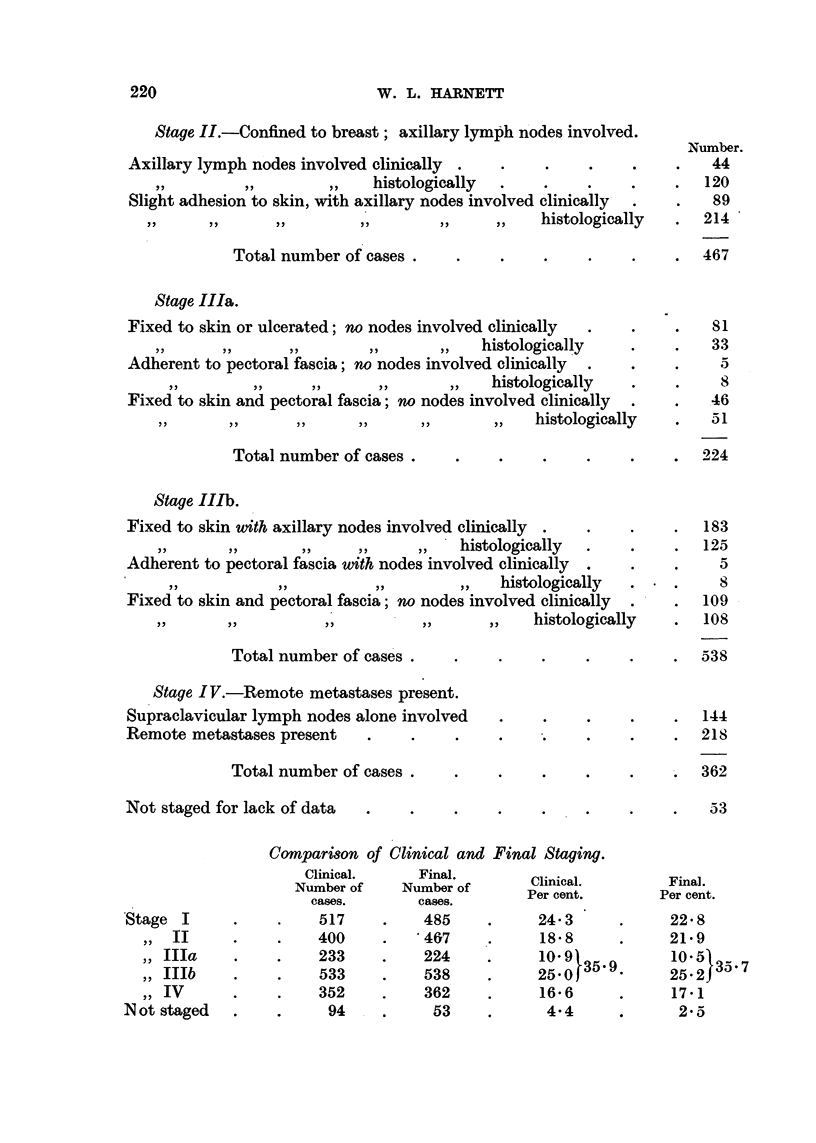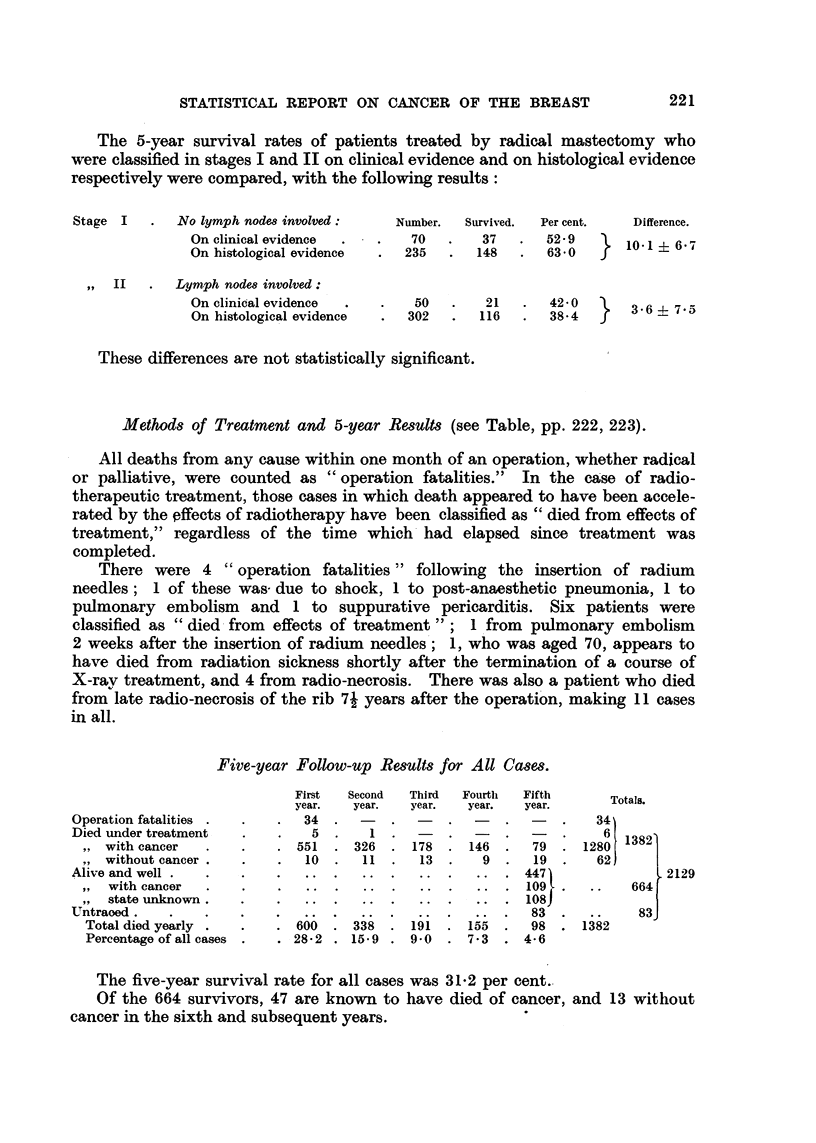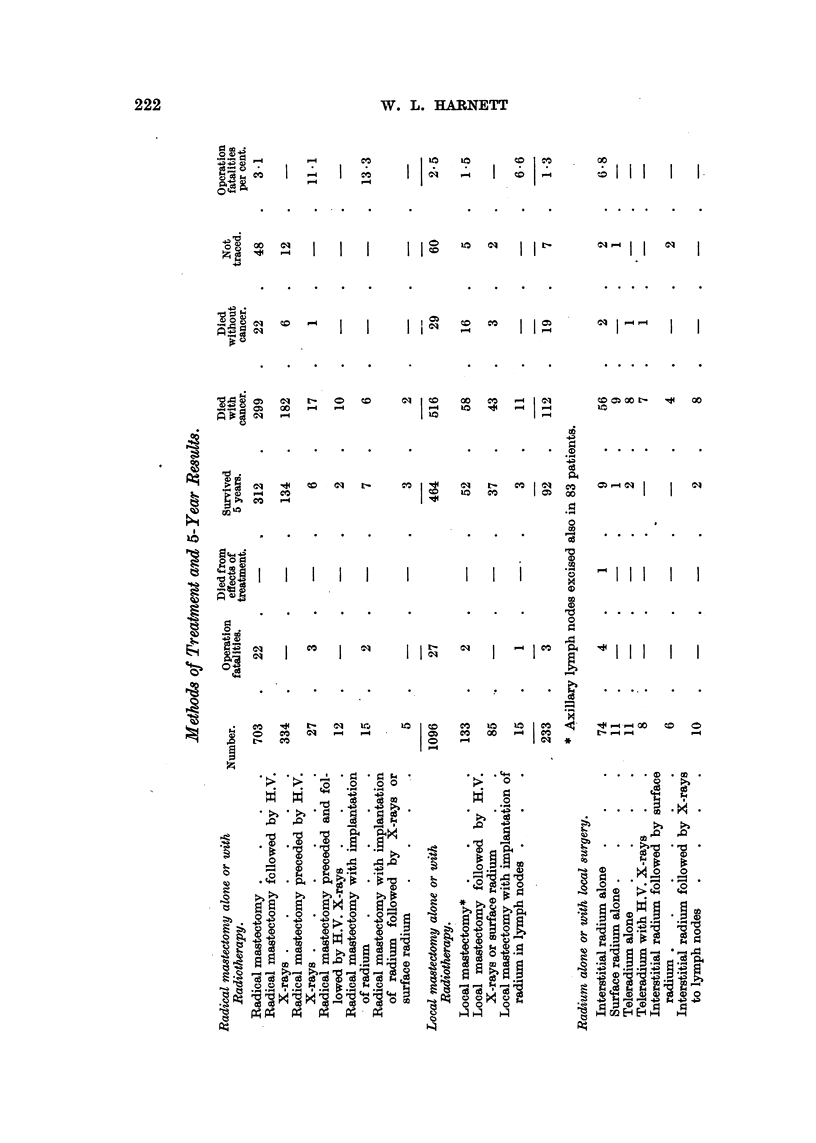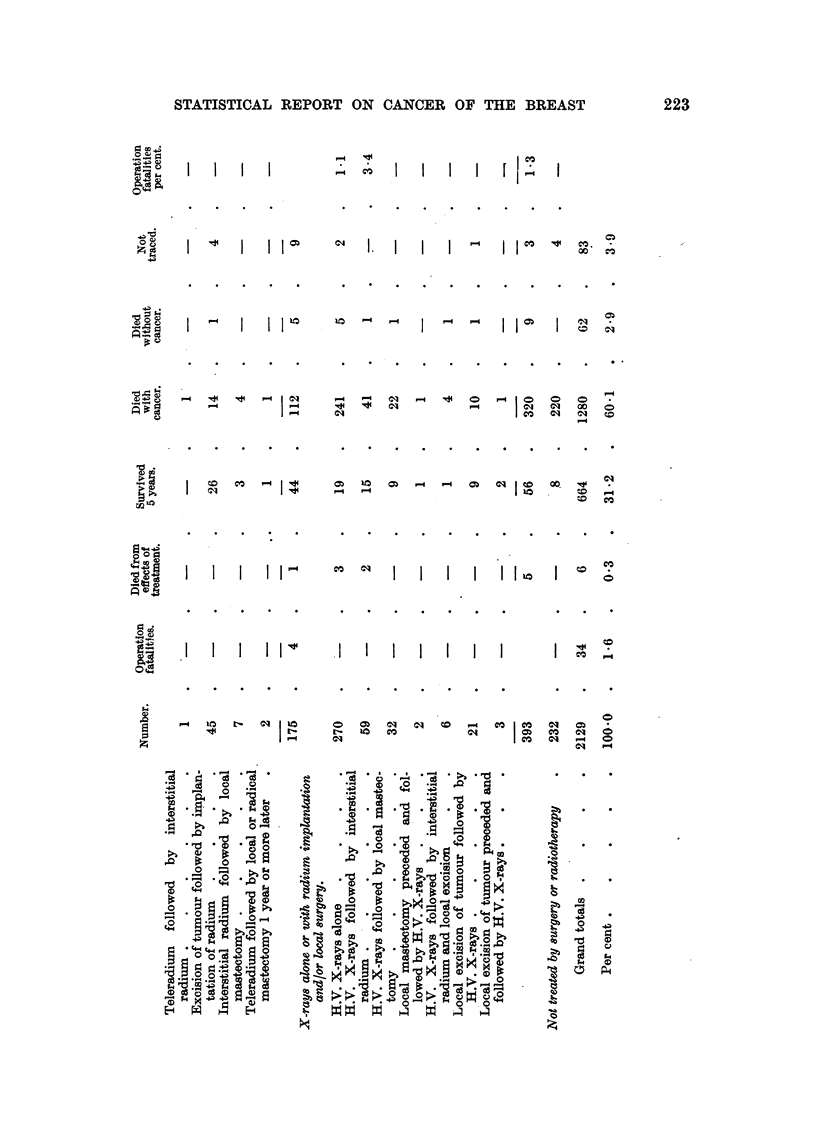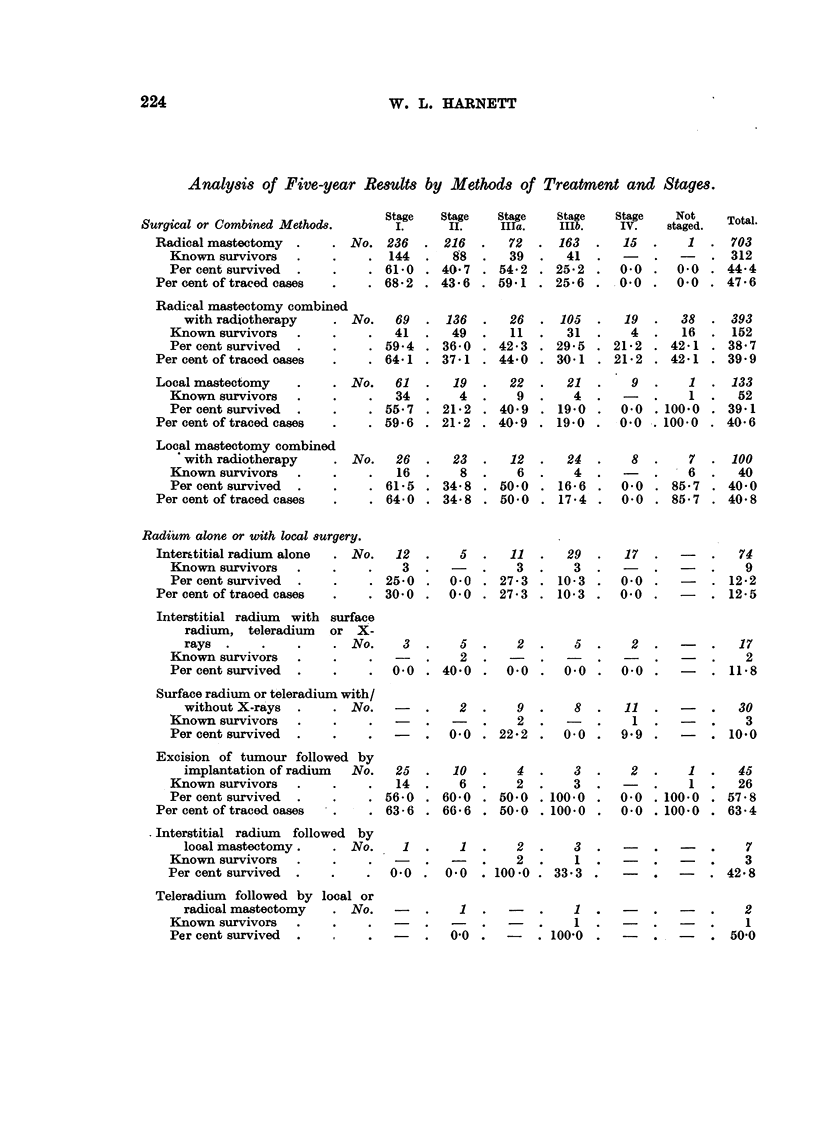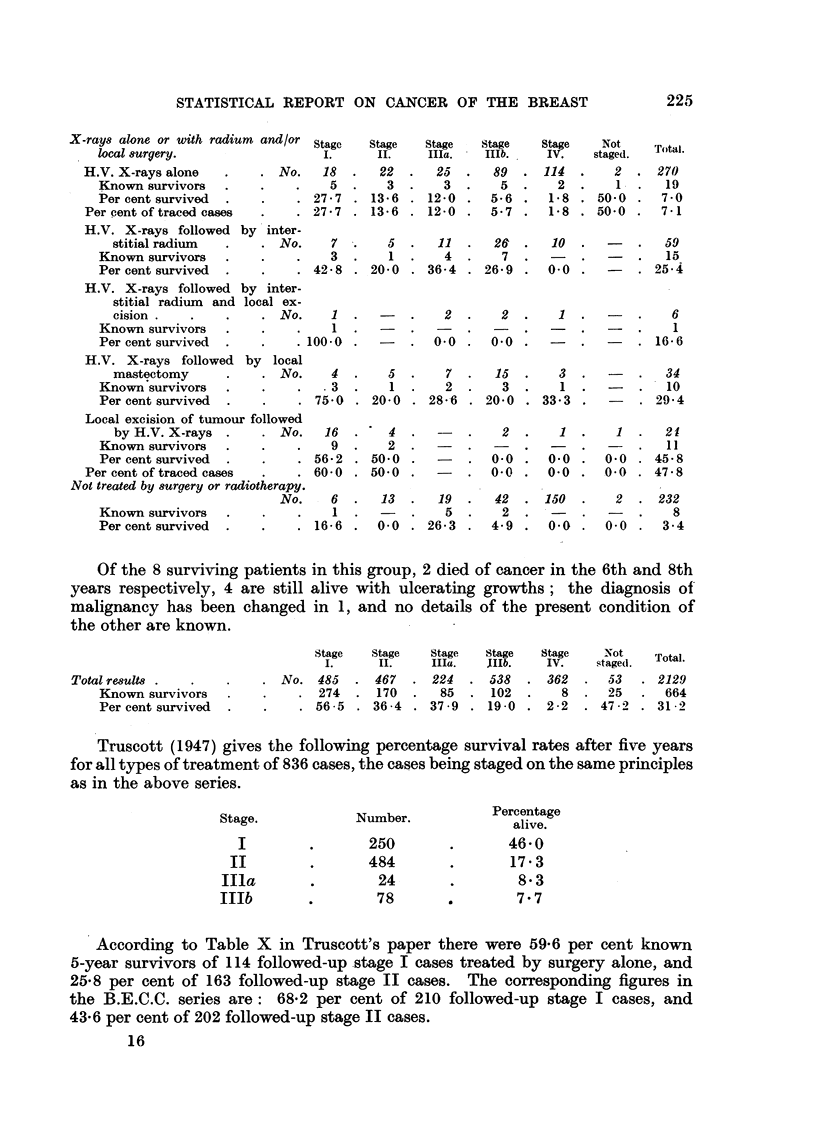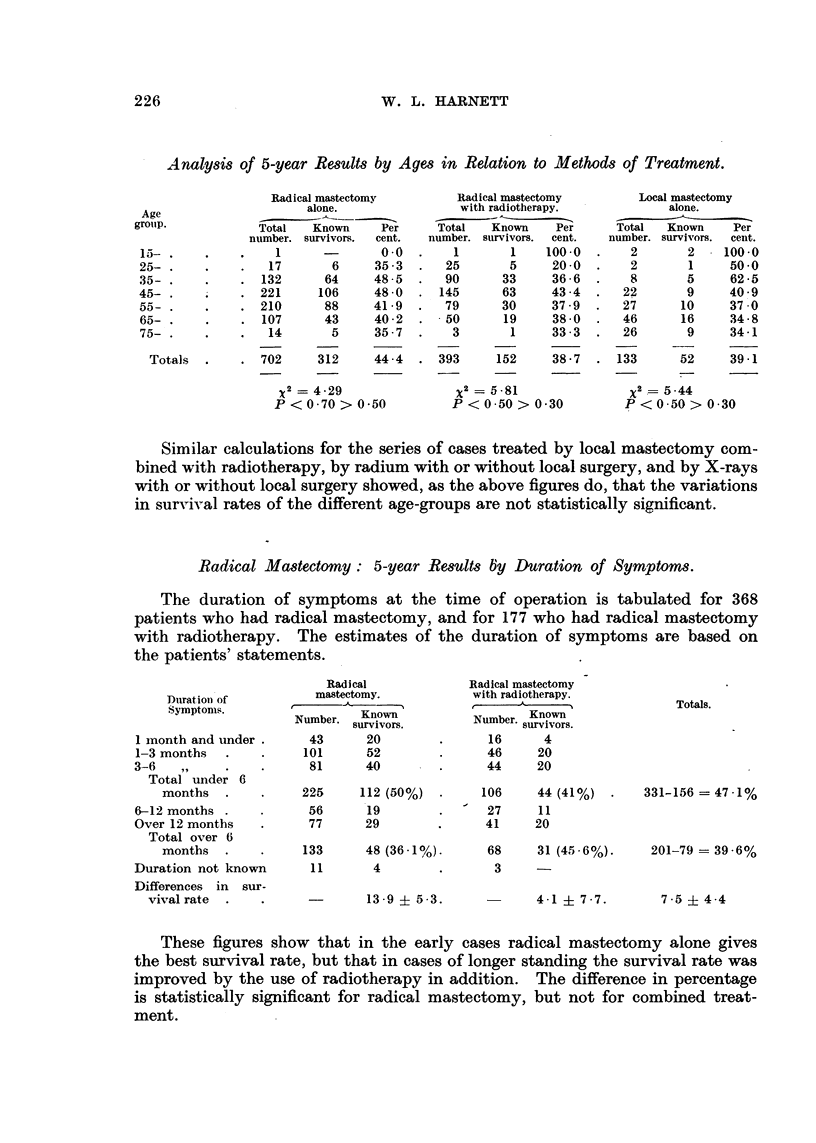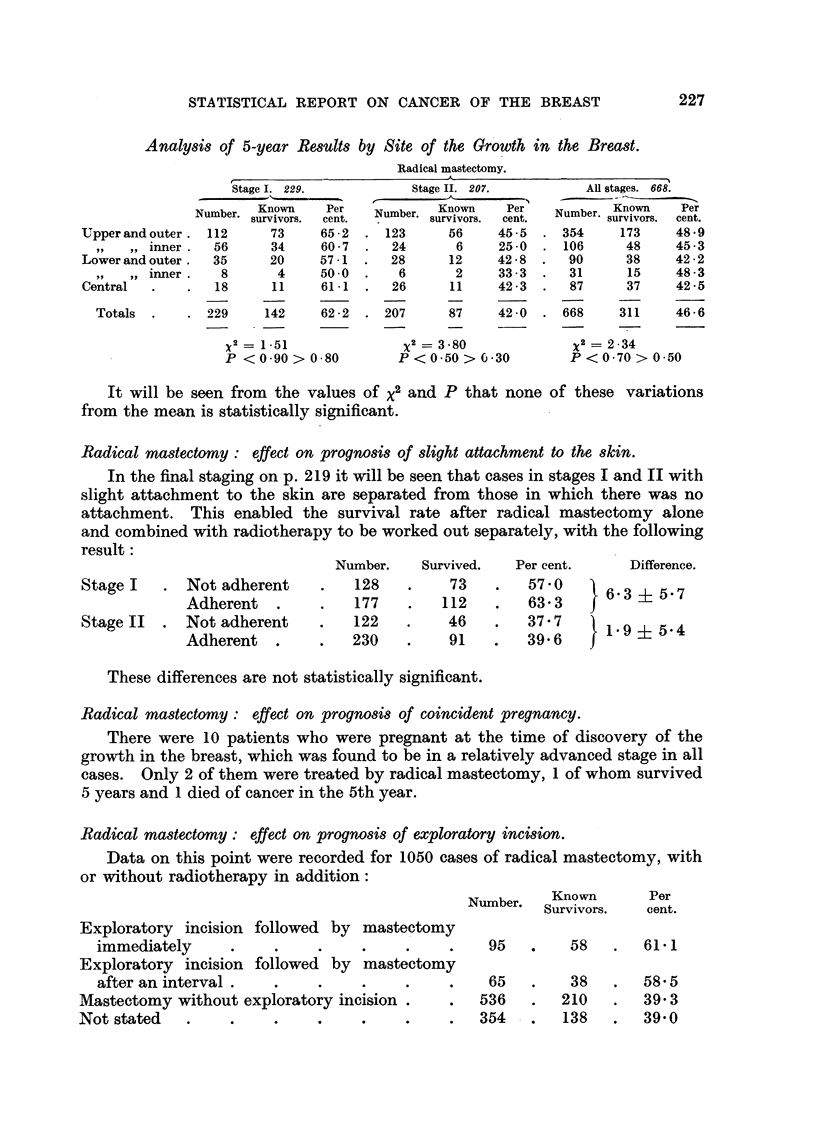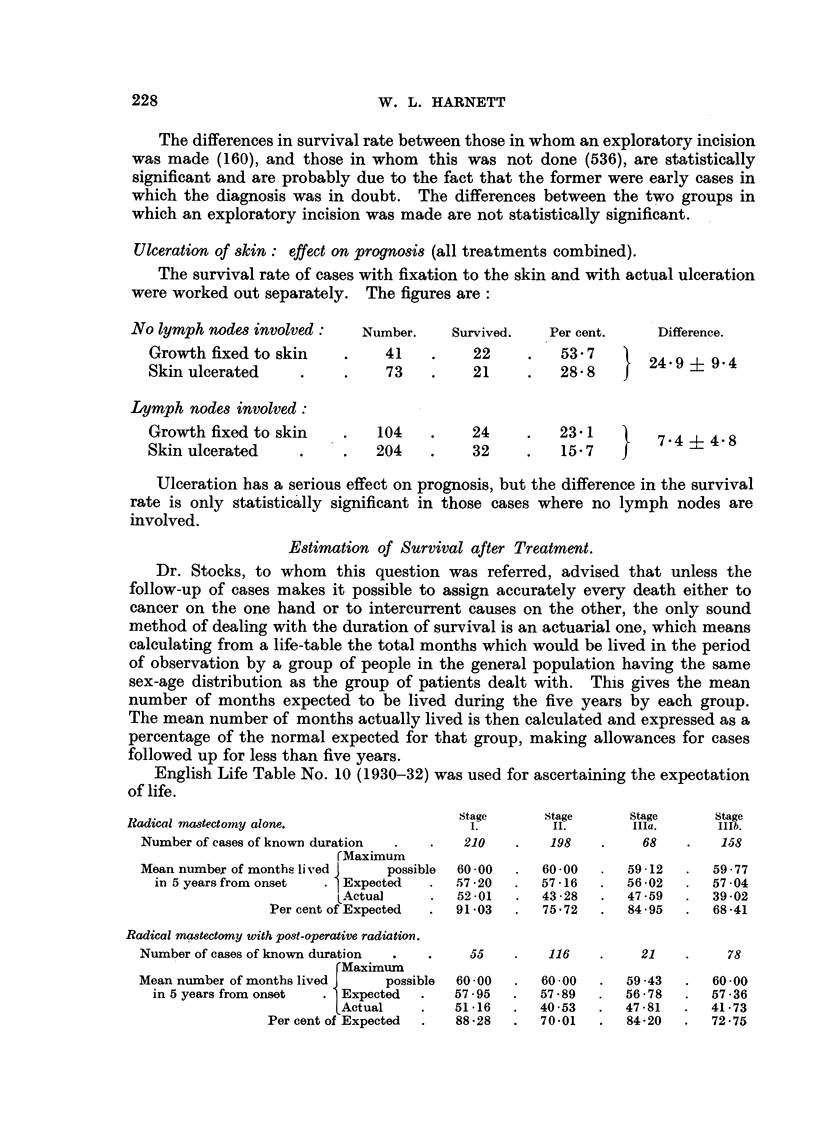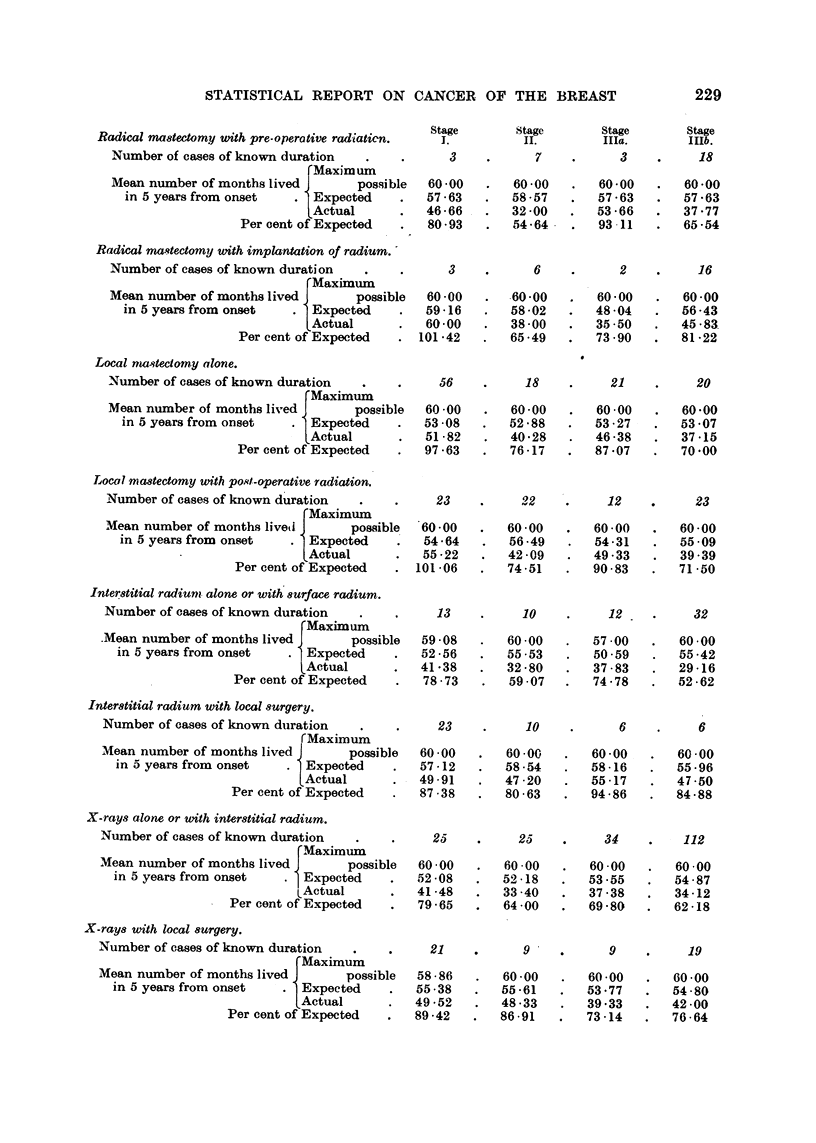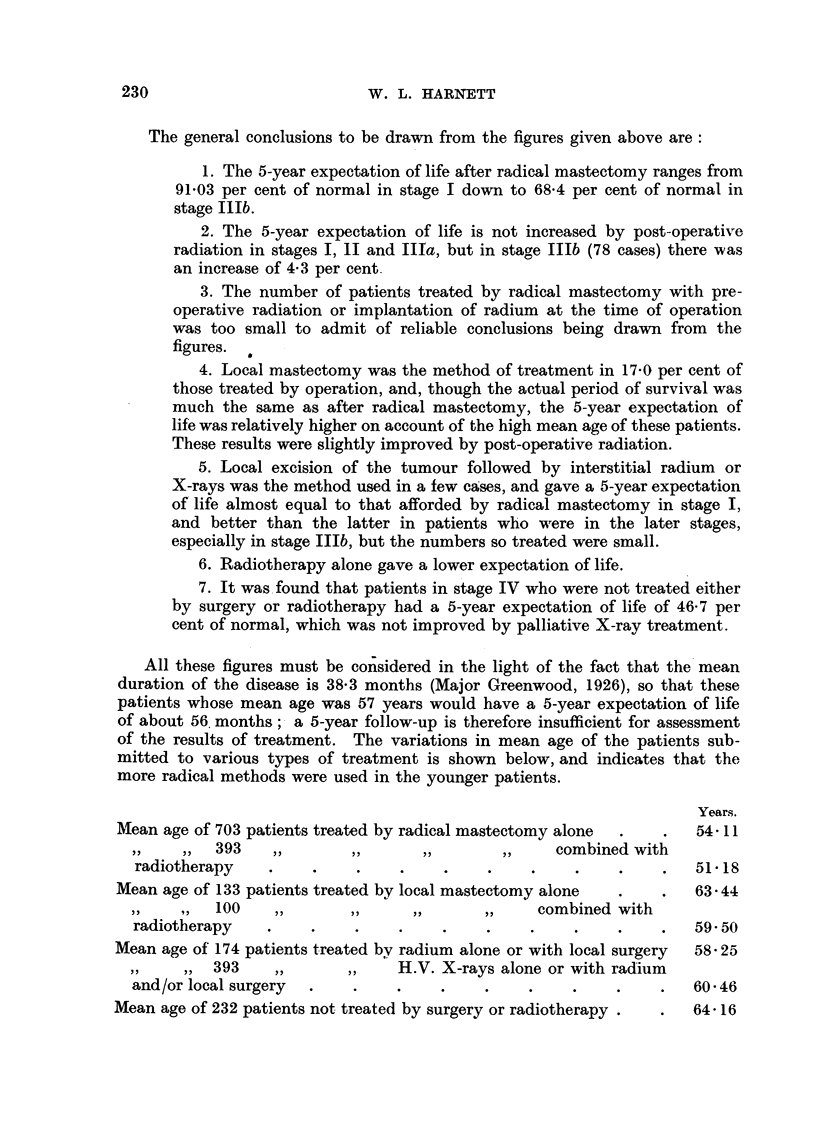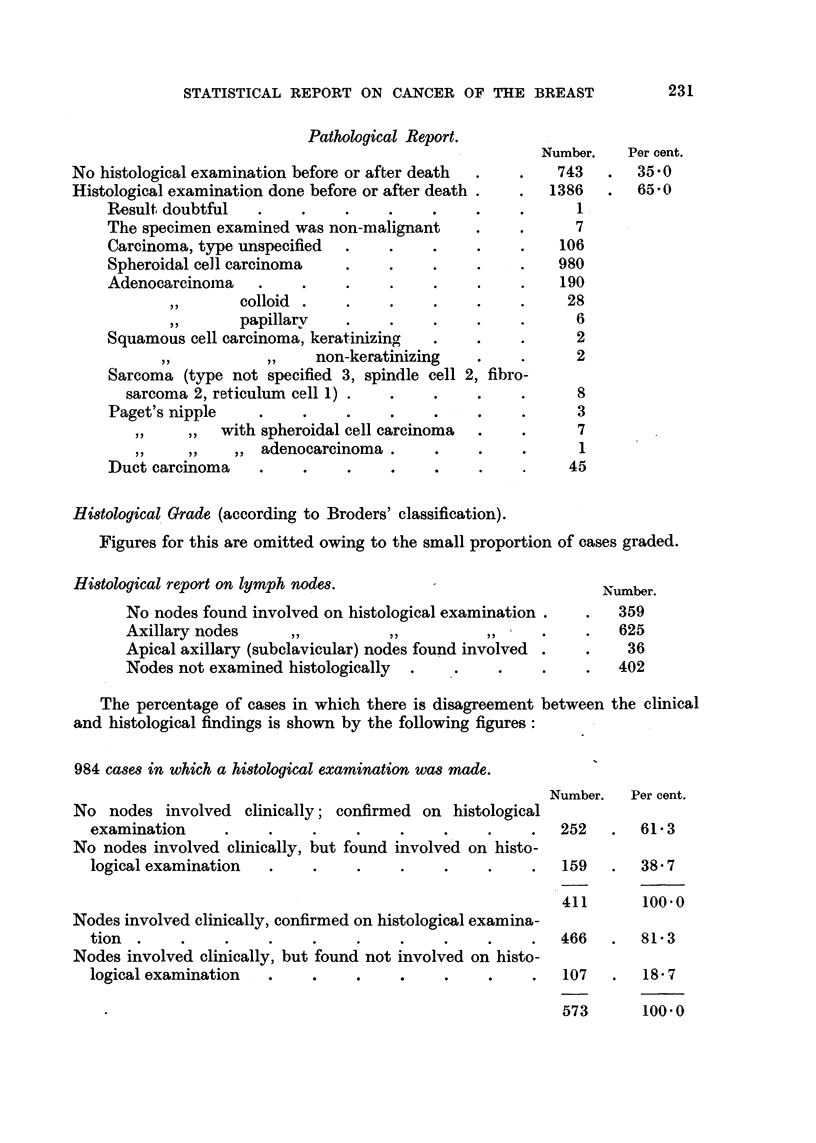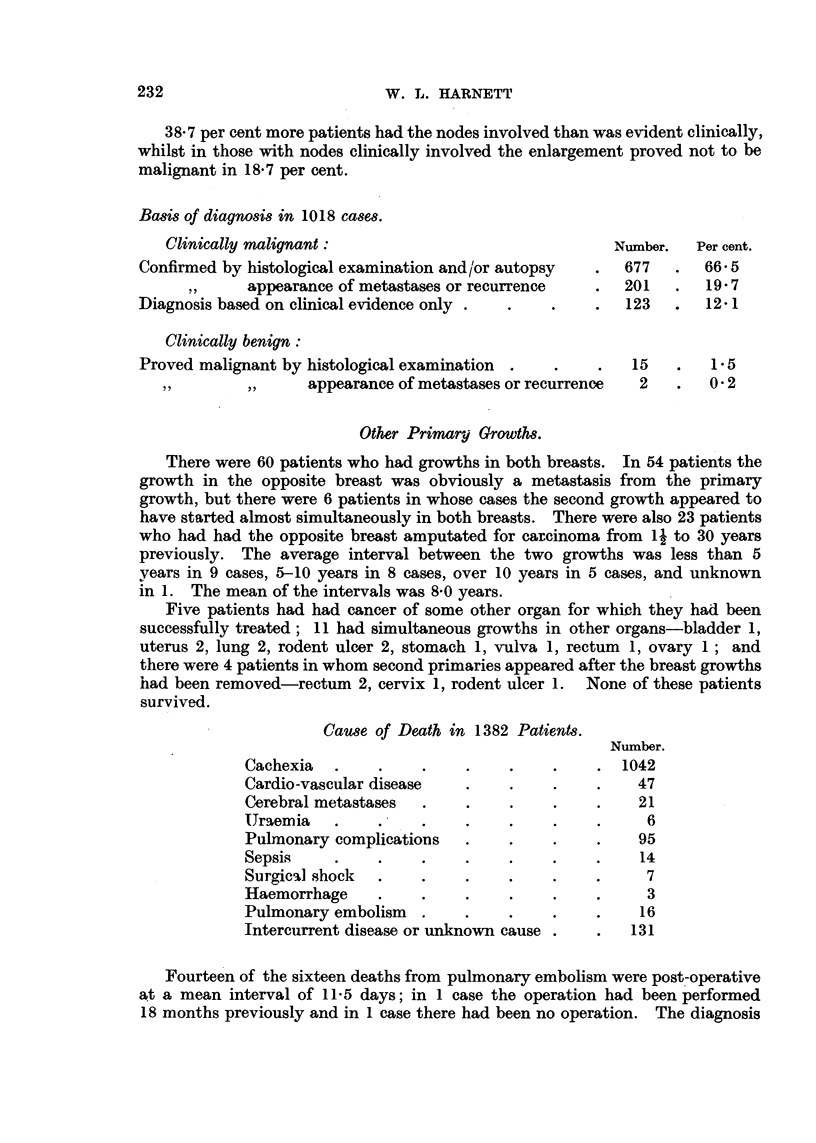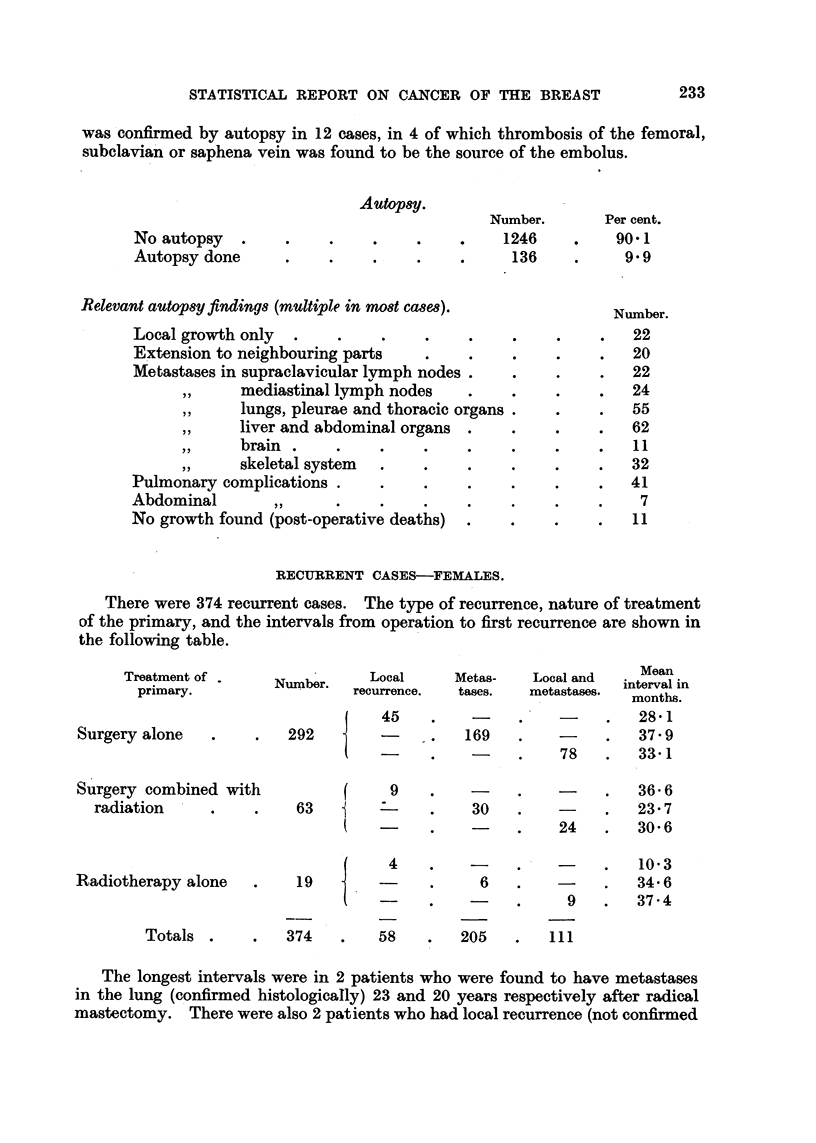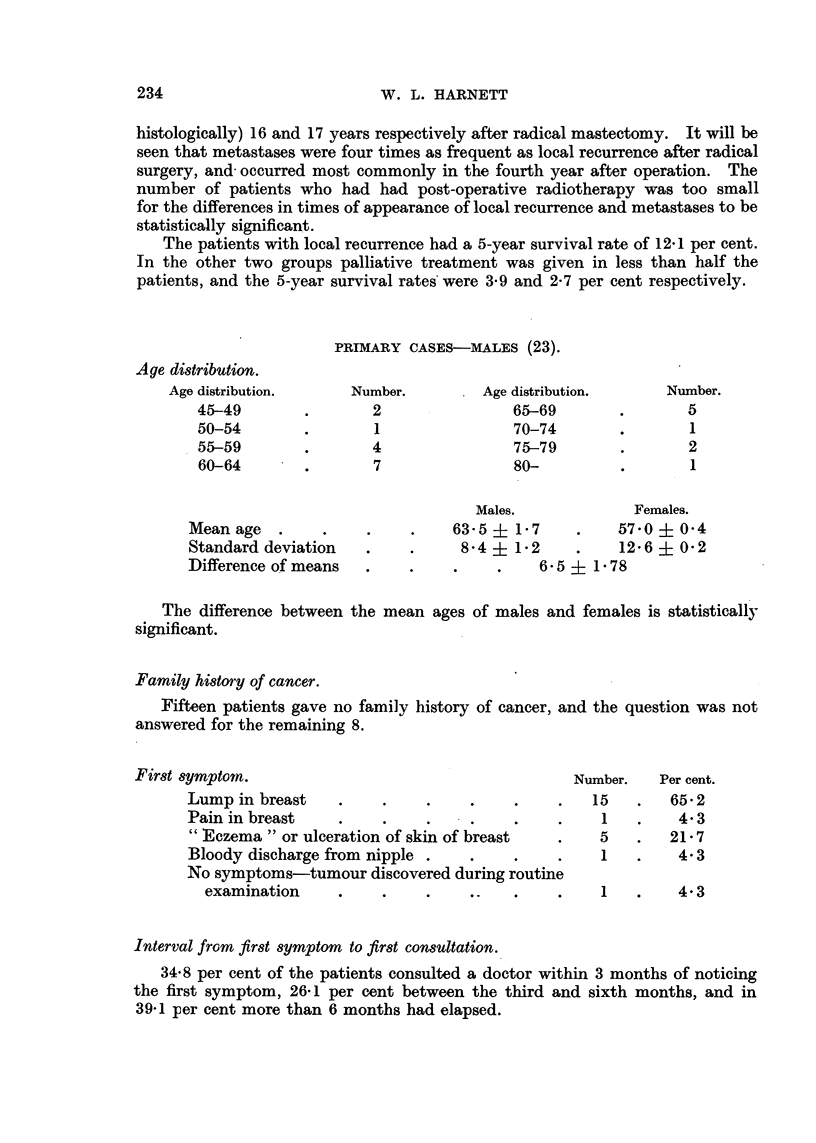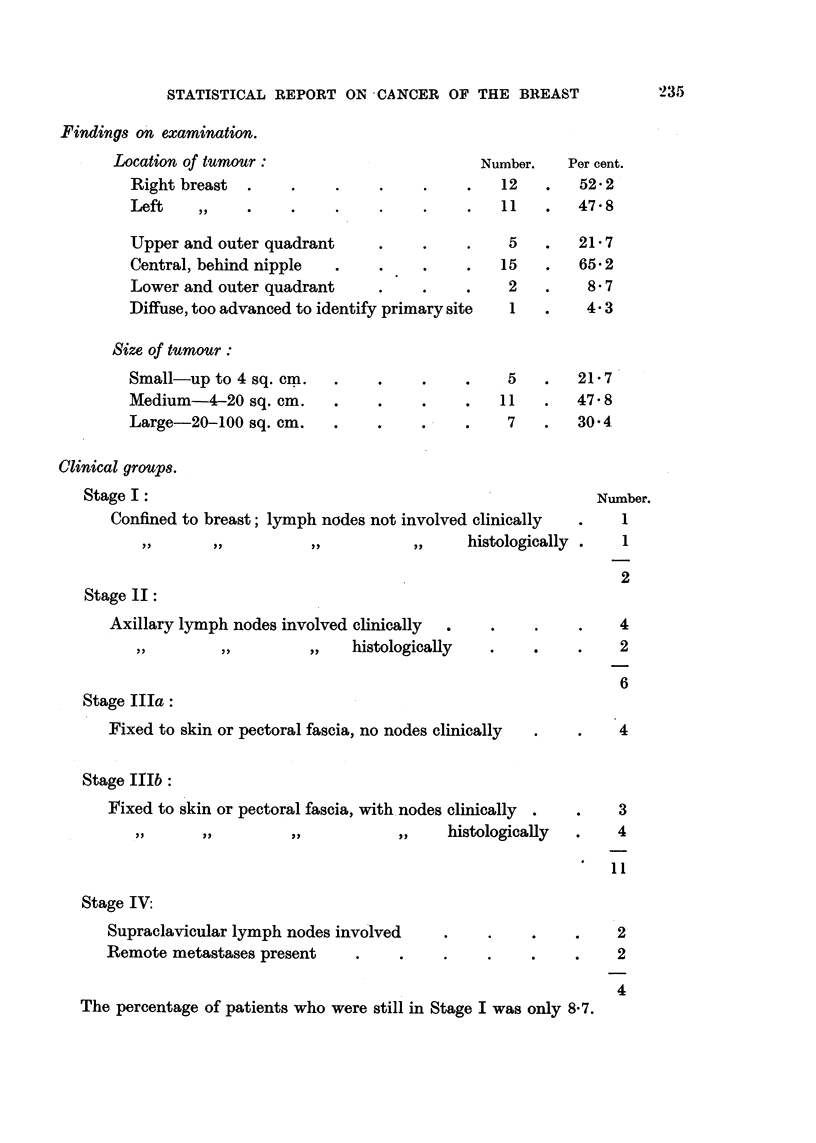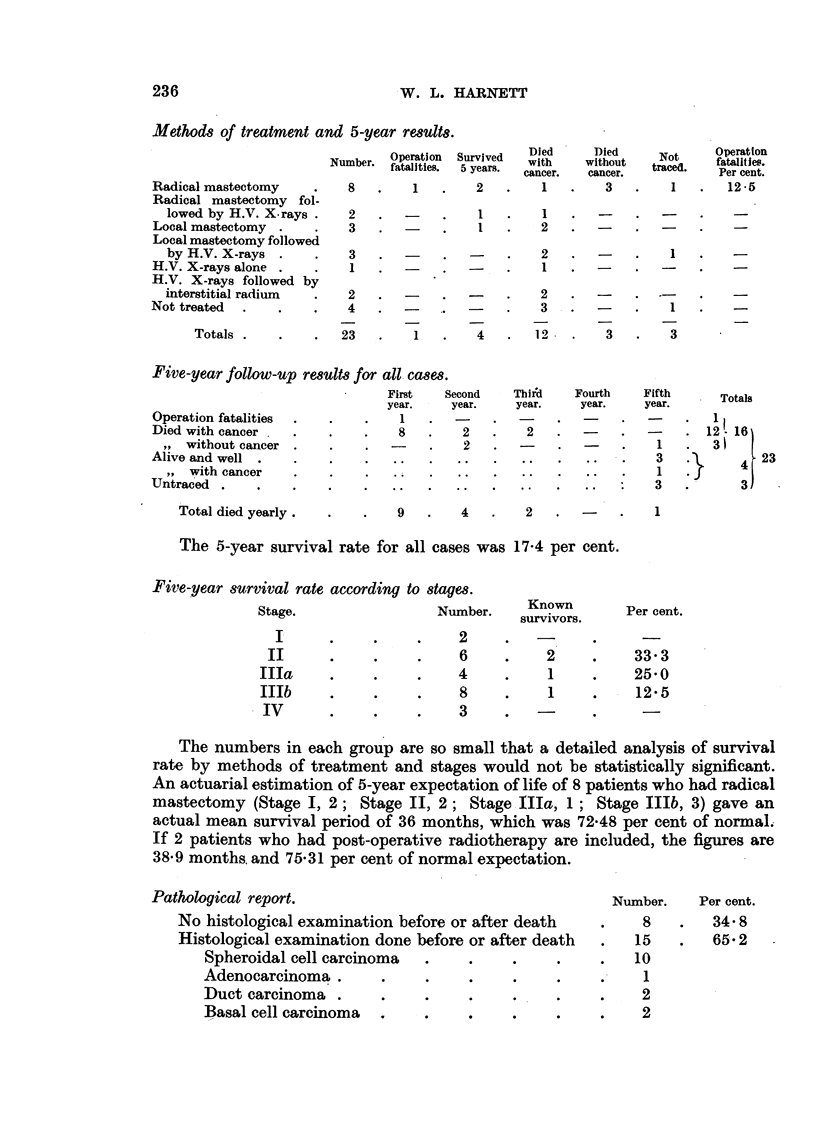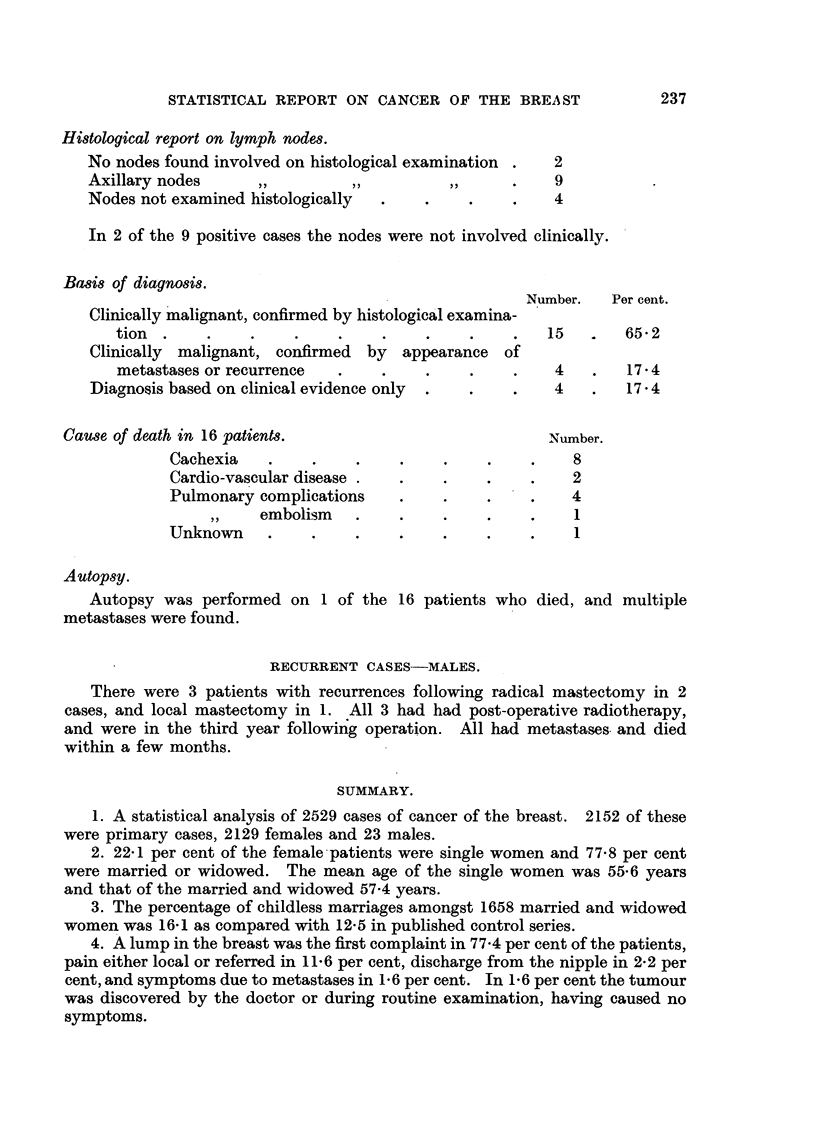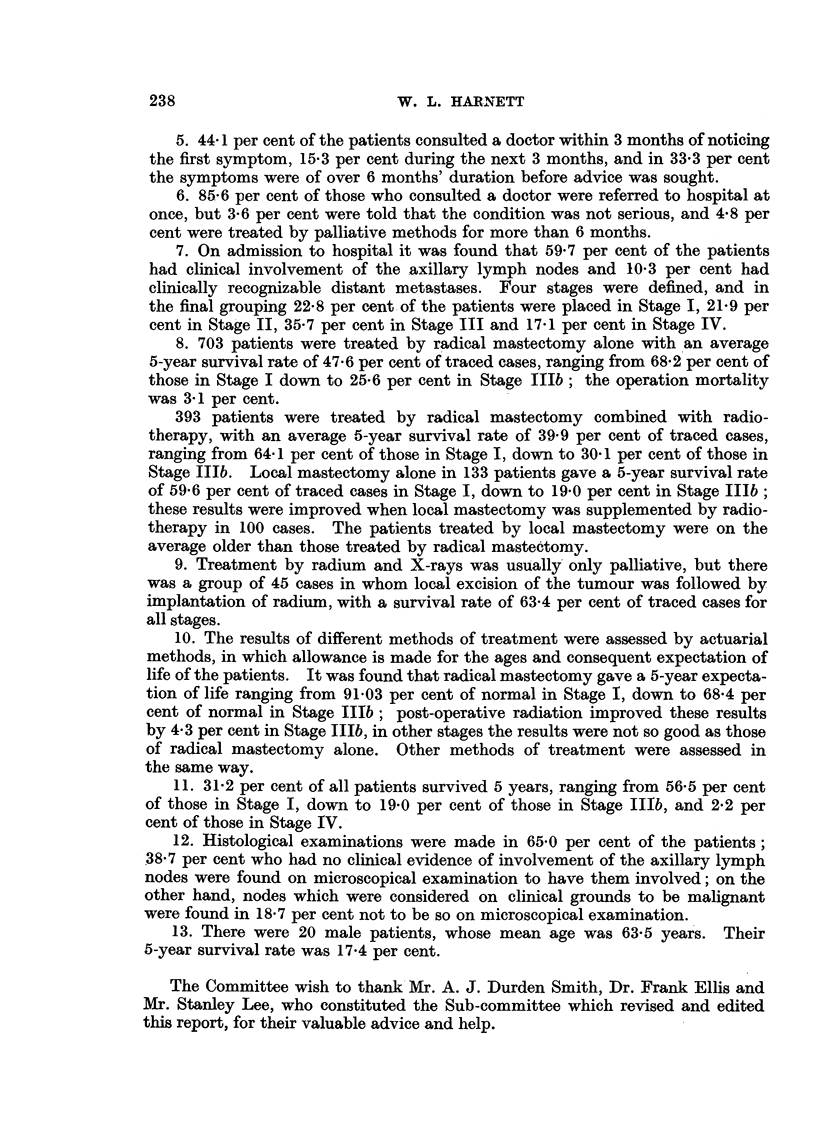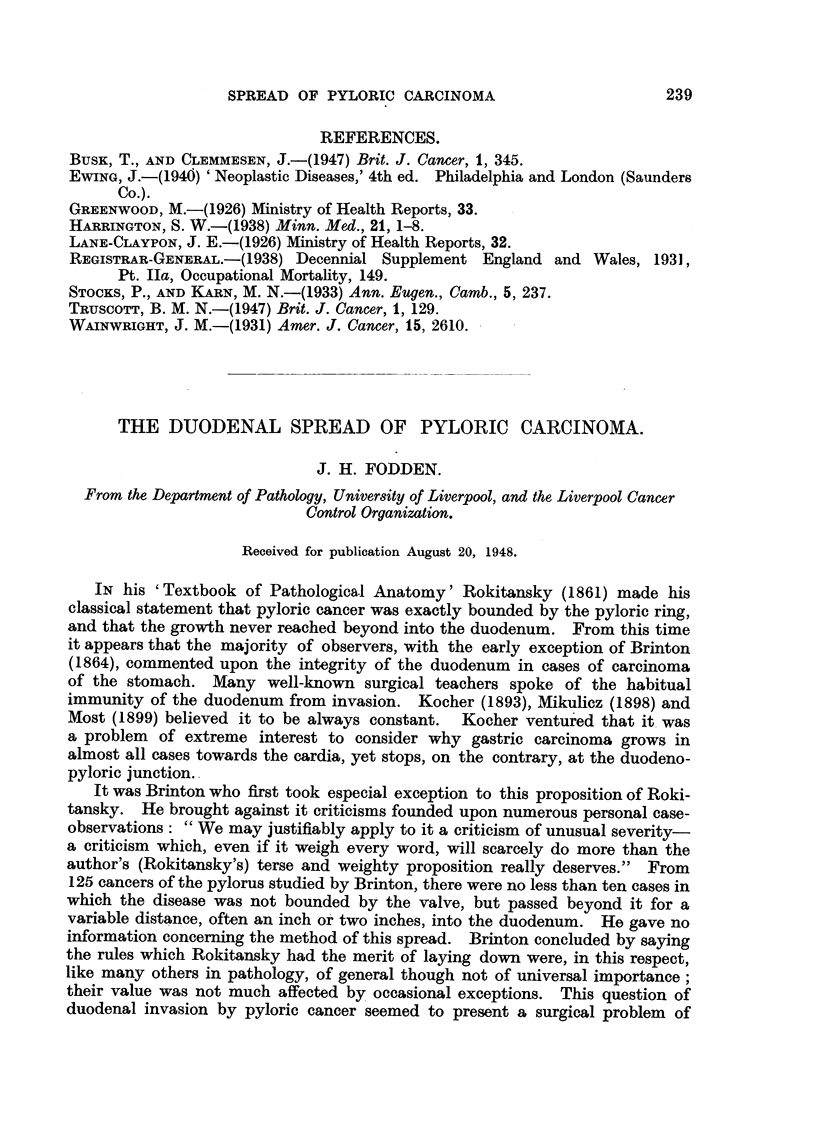# A Statistical Report on 2529 Cases of Cancer of the Breast

**DOI:** 10.1038/bjc.1948.27

**Published:** 1948-09

**Authors:** W. L. Harnett


					
-.STATISTICAL REPORT ON 2529 CASES OF CANCER

OF THE BREAST.
W. L. HARNETT.

Published for the Clinical Cancer Research Committee of

the British Empire Cancer Campaign.

Received for publication July 16, 1948.

IN 1938-39 the Clinical Cancer Research Committee of the British Empire
Cancer Campaign carried out a clinical survey of all cases of cancer seen in the
hospitals, both Voluntary and L.C.C. in the Administrative County of London.
This was done by means of questionnaires, one of which was filled in by the
Registrars in the various hospitals for each patient and returned to the Clinical
Cancer Research Committee for record. The work was interrupted by the
outbreak of war after it had been in progress for 17 months. By this time
15,200 cases of cancer had been registered, of which 2529 (16*6 per cent) were
cancer of the breast. These patients have now been followed up for five years
or more and the records analysed. The full report will be published in due
course by the British Empire Cancer Campaign, but the main findings are here
presented.

There were 2152 primary and 377 recurrent cases. 23 of the 2152 primary
cases were males (1.07 per cent) and 2129 were females (98.93 per cent)-the same
ratio as found in other large series.

PRIMARY CASES--FEMALES (2129).

The percentage of single, married and widowed amongst the 2129 females is
shown below, together with the corresponding figures from Lane-Claypon's
(1926) report on cancer of the breast, in which figures for 508 cancer patients
were compared with 509 controls.

STATISTICAL REPORT ON CANCER OF THE BREAST

Civil State.

British Empire

Cancer Campaign,

2129 cancer cases.      508 c
Number.    Per cent.   Number

471         22.1     .    116
1236         58.0    .    292
422          19.8    .   100

Lane.-Claypon.

?_

Dancer cases.

r.  Per cent.

22-8
57.5
19.7

509 control cases.

Number.   Per cent.

87       17.1
321       63.1
101       19.8

The difference between the percentages of single women in the cancer series
and in the control series, 5-7 + 2.5, is statistically significant. It will be seen
that the percentages in the B.E.C.C. series are almost the same as those in Lane-
.Claypon's cancer series. The Registrar-General (1938) concludes from an analysis
of 13,298 deaths from cancer of the breast that "at each age above 35 single
women suffered considerably higher mortality than others from this cause."

Age

distribution.

15-24 .   .
25-34 .    .
35-39 .    .
40-44 .    .
45-49 .    .
50-54 .    .
55-59 .    .
60-64 .    .
65-69 .    .
70-74 .    .
75-79 .    .
80-84 .    .
85 and over.
Mean age

Standard deviation .

Age Distribution.

Single.

3
19
23
55
61
80
53
57
48
44
17

7
..     4.

55.6 ? 0 6
12 .8 ? 0.4

Difference between mean ages of single and married 57. 4-556 - 1 8+ 0 66.

The mean age is 5-6 years higher than those given by Lane-Claypon (1926)
and Wainwright (1931), due to the fact that the two latter series include only
cases coming to hospital for operation, whereas the B.E.C.C. series includes all
cases seen, whether operable or not. The youngest patient in the series was
aged 15, and survived 5 years after enucleation of a tumour of 3 years' standing,
which proved on histological examination to be an early adenocarcinoma. The
oldest patient was aged 90, and died of recurrence 5 months after amputation
of the breast for an ulcerating growth, proved histologically to be an adeno-
carcinoma.

Single .
Married.
Widowed

Married and

widowed.

1
40
99
147
217
214
219
223
217
141
100
30
10

574 + 0 3
12.5 - 0.2

Total.

4
59
122
202
278
294
272
280
265
185
117

37
14

57 0 i 0.4
12.6 + 0 2

213

W. L. HARNETT

Heredity.

A history of the patient's father having died of cancer of any region was given
by 4.5 per cent of all patients, and of death of the mother from cancer of any
region by 8.3 per cent, from cancer of the breast by 3.0 per cent. 382 patients
gave histories of 452 parents or siblings having suffered from cancer, so that in
70 instances (3.3 per cent) more than one relative was affected. There were
17 instances of both parents having died of cancer amongst 1589 patients of
known family history, or 1 in 93; the expectation of this occurring by chance
is 1 in 151; Stocks and Karn (1933) found the expected frequency of this event
for cancer of all regions to be 1 in 180, the numbers involved being 364 fathers
and 323 mothers. As no information about family history was available for
25.4 per cent of the patients, it is impossible to draw definite conclusions from
the figures.

Relation to the Menopause.

30.4 per cent of the single women and 25.5 per cent of the married had not
reached the menopause, and in 2.6 per cent and 3.4 per cent respectively it was
in progress when the disease first appeared. In 18.7 per cent and 18-3 per cent
the question was not answered. Lane-Claypon (1926) has shown that the mean
age of cessation of the catamenia in a series of 328 patients with cancer of the
breast was not significantly different from that of a control series of 332 women.

Children and Miscarriage.

The percentage of childless marriages amongst 1658 married and widowed
women was 16,1; Lane-Claypon found that 18.4 per cent of 261 women with
cancer of the breast and 12.5 per cent of 280 control patients were childless.
She concluded from a statistical study of the data by Major Greenwood that
"the fertility of the cancer patients is definitely less than that of the control
patients." As no information on this point was available for 13.5 per cent of
patients in our series, it is impossible to say whether or not the figures support
Lane-Claypon's conclusions. For the same reason we can give no reliable figures

Number of     Per cent.

cases. ~Per cent.
cases.

Lump in breast    .    .    .    .    .    1649    .    77.4
Pain in breast    .    .    .    .    .     213    .    10.0
"Eczema" of skin of breast  .    .    .     57     .     2.7
Retraction of nipple   .    ..               41    .     2.0
Referred pain     .    .    .    .    .      34    .     1 6
Symptoms due to.metastases .     .    .      34    .     1-6
Bloody discharge from nipple .   .    .      26    .     1-2
Discharge from nipple  .    .    .    .      20    .     1.0
Lump in axilla    .    .    .    .    .      18    .     0 8
Loss of weight and' general weakness  .       2    .     0.1
No symptoms. Tumour discovered during

routine examination  .    .    .    .      34    .     16

Not stated  .     .    .    .    .    .       1    .     0.05

214

STATISTICAL REPORT ON CANCER OF THE BREAST              215

of the proportion of women with cancer of the breast who had or had not nursed
their children. Lane-Claypon found that 14.6 per cent of the children of women
who afterwards developed cancer of the breast were not nursed anid 7.4 per cent
of the children of those in the control series-a statistically significant difference.

First Symptom.

A lump in the breast was the first symptom complained of in 77.4 per cent
of the patients, pain in the breast in 10.0 per cent, and other symptoms as shown
in the table (see p. 214).

The 34 patients in whom the first symptoms were those due to metastases
were found to have the following:

Number of

cases.

Symptoms due to enlarged axillary nodes  .    .    .     2

,,  ,,    ,,   supraclavicular nodes  .   .     3
,,  ,,    ,,   lymph nodes elsewhere.     .     1
,,   ,,  metastases in vertebral column  .  .    13
,,  ,,    ,,      liver  .    .     .    .    5
,,  ,,    ,,      lung   .    .     .    .    3
,,  ,,    ,,      brain  .    .     .    .    3
,,  ,,    ,,      skull   .    .    .    .    2
,,  ,,    ,,      femur .     .     .    .    2

Bloody discharge from the nipple was the first symptom in 26 patients.
The histological reports on these tumours were: spheroidal cell carcinoma 8,
duct carcinoma 7, adenocarcinoma 4, carcinoma, type not specified 1, squamous
cell carcinoma 1, not examined histologically 5.

Interval from First Symptom to First Consulting a Doctor.

Number of

Per cent.
cases.

1 month and under .     .    .    .     508S

1-2 months    .    .    .    .    .     266      .    44-1
2-3   ,,      .    .    .    .     .    164J

3-4   ,,      .    .    ....            1173

4-6   ,,      .    .     .    ..         20Sf          15*3
4-6                          ~~~~~~208J

6-9    ,,     .    .    .    .     .    136
9-12  ,,      .    .    .    .     .    190
12-18  ,,      .    .    .    .    .      83

18-24  ,,      .    .    .    .    .     119           333
24-48  ,,      .    .     .    .    .     85

Over 48 months.      .    .    ..         97J

Not stated .    .    .    .    .    .     156     .      7.3

These figures show that there was serious delay on the part of 48-6 per cent
of the patients in seeking medical advice, and in. .6 per cent the interval was
2-4 years or more.

W. L. HARNETT

Advice and Treatment before Admission to Hospital.

Number of cases.

No doctor consulted prior to coming to hospital .  .     293
Referred to hospital without delay  .   .    .    .     1472
Advised to go to hospital, but delayed going there  .     99
Treated symptomatically for periods up to 3 months

before reference  .    .    .    .    .    .    .       33
Treated symptomatically for periods of more than 3

months before reference .   .    .    .    .    .       88
Reassured and told condition not serious  .  .    .       66
Not stated         .     .    .    .    .    .    .       78
Refused the treatment advised  .   .    .    .    .       22

Of the 1836 patients who consulted a doctor, 85.6 per cent were referred to
hospital at once, though 5.4 per cent of these delayed in following this advice or
perhaps had to wait some weeks for a vacant bed; 1.8 per cent were kept under
observation and symptomatic treatment for periods up to 3 months, and 4.8 per
cent for longer periods, but many of the patients in the latter group were in an
advanced stage of the disease, or were unsuitable for operative treatment by
reason of poor general condition. 3.6 per cent of the patients were reassured
and told that there was nothing serious the matter. Only 1.0 per cent refused
the treatment advised at hospital.

Location of

tumour.

Left breast .
Right breast
Both breasts
Not stated .

Findings on Examination in Hospital.

Number of

cases.

~.  ~..   .   ~1114
? ?.  . ?  .    1005

6

? .    .      .        64

? ?     ?     ?         4.

Per cent.

52*3 ? 1*09
47*2

0 3
0*2

Busk and Clemmesen (1947), investigating the site of 4139 Danish cases, found
that the proportions were 111 on the left to 100 on the right-almost exactly the
same figures as in our series -and that the difference was statistically significant.
Ewing (1940) states that the disease appears to originate simultaneously in
both breasts in 1.5 per cent, Harrington (1938) found simultaneous carcinoma
in 1.0 per cent and bilateral carcinoma occurring at different times in 4-5 per
cent of 4628 cases. In the present series 60, or 2-8 per cent, were found to have
growths in both breasts, but 54 of these were considered to be metastases from
a pre-existing primary in the other breast, and 6 to be examples of simultaneous
carcinoma.

Site in breast.                 N

Upper and outer quadrant    .

inner    ' ,
Central, behind nipple

Lower and outer quadrant    .

inner     ,,   .    .

Diffuse, too  advanced  to identify the

primary site    .    .    .
Not stated   ..             .

rumber of cases.

916
283
235
204

94
324

74

Per cent.

43.0
13.3
11.0

9 6
4.4

15 2
3-5

216

STATISTICAL REPORT ON CANCER OF THE BREAST                 217

There was 1 patient who had 2 separate growths in the upper inner and lower
outer quadrants respectively, making a total of 2130 tumours.

Size of tumour.                      Number ofs.   Per cent.

caseS.

Small-up to 4 sq. cm    .    .    .     .     216    .     10.1
Medium     -4-20 sq. cm  .   .    .     .     717     .   33- 7
Large-20-100 sq. cm     .    .    .     .     752    .    35-4
Massive-over 100 sq. cm      .    .     .     193     .     9.1
Not stated   .    .     .    .    .     .     251     .    11-8

Ulceration of                              Number of     Per cent.

Per cent.
skin.                                    cases.

Present    .      .     .    .    .     .     439    .    20.6
Not present .     .     .    .    .     .    1614    .    75.8
Not stated   .    .     .    .    .     .      76    .      3 6

Skin Metastases. Clinical Involvement of Lymph Nodes.

Number of     Per cent.

cases.

Skin nodules present    .    .    .     .     245    .    11-5

,,  ,,  not present   .       ..        1789     .    84-1
Not stated   .    .     .    .    .     .      95    .     4.5

Axillary lymph nodes involved:

Unilateral .    .     .    .    .     .    1195    .    56-1
Bilateral  .    .     .    .    .    .       76    .     3- 6
No lymph nodes involved      .    .     .     812    .    38- 2
Not stated   .    .     .    .    .     .      46    .     2 2

Supraclavicular nodes also involved:

Unilateral .    .     .    .    .     .     189    .     9.0
Bilateral  .    .     .    .    .    .       31    .     1.5

These figures include all cases in which the lymph nodes were palpable; in
many of them other complications such as fixation to skin or distant metastases
were also present.

Remote Metastases.

Number of

Per cent.
oases.

None found on clinical examination  .  1075

,,  ,,   radiological                   1824   .     85 7

examination     749

Metastases present .     .    .    .    219         .      10.3
Not stated    .    .     .    .    .     86         .      4*0

W. L. HARNETT

Sites of Metastases in 219 Patients (Multiple in Many Cases).

Mediastinal lymph nodes   .

Lymph nodes, other than regional nodes
Skin and subcutaneous tissues
Lungs and pleurae  .

Liver and abdominal organs.
Brain     .    ..

Skull, spine, ribs and sternum
Bones of pelvis .  .

Bones of extremities  . .    .

27

7
? 6

86
49

8
69
24
30

This table does not include metastases in the supraclavicular lymph nodes,
the figures for which are shown above.

Condition of the Opposite Breast.

I
A cancerous growth was present  .    .
Opposite breast had been amputated pre-

viously for cancer  .

Nodular mastitis or cysts present - .

Opposite breast has been treated surgically

or by radiotherapy for mastitis
Normal .     .    .    .
Not stated  .    .    .

Number of cases.

60

23
74

10
1866

96

Of the 60 cases with growths in both breasts, 6 were considered to be true
simultaneous growths, and 54 to be metastases from a primary in the other
breast. The 23 cases in which the opposite breast had been amputated for
cancer previously may have been either recurrences or second primaries.

General Condition.

IN
Obese; general condition not noted .
Good condition; no weight loss

Fair condition; moderate weight loss (up

to 2 st.)  .    .    .

Poor condition; considerable weight loss

(over 2 st.)    .    .
Emaciated    .    .    .
Moribund     .    .    .
Not stated   .    .    .

Other Co-existing Diseases.
Was suffering from diabetes    .

,, ,,    ,, cardiovascular disease

Y      ,, ,,  ,, pulmonary tuberculosis .
,, ,      ,,   ,mental affection (usually

senile dementia)- .

Number of cases.

13
1203

468
189

33
27
196

23
71

5

26    .     1.2

218

Per cent.

2 8

1'1
3.5

0'5
87-6
4.5

Per cent.

0'6
56*5

21*9

8-9
1 *6
1*3
9'2

1-1
3.4
0.2

STATISTICAL REPORT ON CANCER OF THE BREAST

Clinical Stages.

The cases were grouped into 4 clinical stages according to the presence or
absence of clinical involvement of lymph nodes, invasion of adjacent tissues and
distant metastases, as follows:

Stage I: Growth confined to breast; no involvement of axillary lymph
nodes nor infiltration of skin or muscles.

Stage II: - Growth confined to breast; axillary lymph nodes involved,
but no infiltration of skin or muscles.

Stage IIIa: Growth infiltrating skin or muscle, or both, but no in-
volvement of axillary lymph nodes.

Stage IIIb : As in IIIa, but axillary lymph nodes involved.

Stage IV: Remote metastases present. Cases with involvement of
the supraclavicular lymph nodes were placed in this stage.

Not staged for lack of data.

Arranged in accordance with this system the numbers in each stage were:

Number of

cases.     Per cent.

cases.

Stage I      .       .   .    517    .    24 3

,, II  .    .    .    .   400    .    18 8
,, IIIa  .  .    .    .   233    .    10.9
,, IIIb  .    .    .   .    533    .    25.0

,, IV    .    .    .   .    352    .    16*6

TV                ~~~~~~352  . 16.6

Not staged .    .    .    .    94    .     4.4

In the case of patients who underwent operation, or died soon after admission,
the operation and pathological findings where available were used for correcting
the staging. Patients not operated on, or who did not die in hospital, were
placed in the appropriate stage according to the clinical evidence of extension of
the growth and the presence of metastases. The table below indicates when the
staging is based on clinical evidence alone and when on histological findings.

Stage I.-Confined to breast; lymph nodes not involved or doubtful.

Number.

No lymph nodes involved clinically .  .  .    .    .   .    .   95

,,     ,,    ,,   histologically  .  .    .    .   .    .  111
Involvement of lymph nodes doubtful clinically  .  .    .   .    6
With slight adhesion to skin; no lymph nodes involved clinically.  .  118

~,,  ,,  ,,  ,,     ~~, ,,      histologically  .  148
,,  ,,     ,, invl61vement of lymph nodes doubtful

clinically  .  .   .    .    .   .    7

Total number of cases .

219

485

220                          W. L. HARNETT

Stage II.-Confined to breast; axillary lymph nodes involved.

Number.

Axillary lymph nodes involved clinically .  .    .    .    .    .   44

,,        ,,        ,,   histologically  .   .    .     .    .  120
Slight adhesion to skin, with axillary nodes involved clinically  .  .  89
~,,  ,,  ,,  ,,       ,,     ,,  ~histologically  .  214

Total number of cases                    .     ..      467
Stage liIa.

Fixed to skin or ulcerated; no nodes involved clinically  ..  .     81

histologically   .     .   33
Adherent to pectoral fascia; no nodes involved clinically  .  .  .   5

histologically  .     .    8
Fixed to skin and pectoral fascia; no nodes involved clinically  .  .  46

,,    ,,      ,,     ,,     ,,       ,,   histologically  .   51

Total number of cases .   .    .    .    .    .     .  224
Stage IIIb.

Fixed to skin with axillary nodes involved clinically  .  .  .  .  183

histologically  .   .    .  125
Adherent to pectoral fascia with nodes involved clinically  .  .  .  5

histologically  .  .      8
Fixed to skin and pectoral fascia; no nodes involved clinically  .  .  109

~~,,  ,,  ,,        ,,     ,,   ~histologically  .   108

Total number of cases .   .    .    .    .     .    .  538
Stage IV.-Remote metastases present.

Supraclavicular lymph nodes alone involved  .    .    .    .    .  144
Remote metastases present   .    .    .    .     .    .    .    .  218

Total number of cases .   .    .    .    .     .    .  362
Not staged for lack of data  .   .    .    .     .    .    .    .   53

Comparison of Clinical and Final Staging.

Clinical.    Final.       Clinical.       Final.

Number of    Number of     Per cent.       Per cent.

cases.   cases.   ~~Per cent.     Per cent.
cases,      cases.

Stage I      .    .    517    .    485    .     24 3      .     22* 8

,, II    .    .    400    .    467     .     18 8     .     21 9
,, IIIa    .    .    233    .    224    .     10 91          105

,, IIIb  .     .   533     .    538    .     250J35'9-      25    35'7
,, IV      .    .   352     .    362    .     16*6      .     17*1
Not staged   .    .     94    .     53     .     4.4      .      2*5

STATISTICAL REPORT ON CANCER OF THE BREAST

The 5-year survival rates of patients treated by radical mastectomy who
were classified in stages I and II on clinical evidence and on histological evidence
respectively were compared, with the following results:

No lymph nodes involved:

On clinical evidence

On histological evidence

,, II   .   Lymph nodes involved:

On clinical evidence

On histological evidence

Number.

70
235

Survived.   Per cent.

37     .    52- 9
148     .   63'0

}

Difference.

10-1 i 6'7

50      .    21         42 0        36     75
? 302      .    116   .    38.4

These differences are not statistically significant.

Methods of Treatment and 5-year Results (see Table, pp. 222, 223).

All deaths from any cause within one month of an operation, whether radical
or palliative, were counted as "operation fatalities."  In the case of radio-
therapeutic treatment, those cases in which death appeared to have been accele-
rated by the effects of radiotherapy have been classified as "died from effects of
treatment," regardless of the time which had elapsed since treatment was
completed.

There were 4 "operation fatalities" following the insertion of radium
needles; 1 of these was- due to shock, 1 to post-anaesthetic pneumonia, 1 to
pulmonary embolism and 1 to suppurative pericarditis. Six patients were
classified as "died from effects of treatment "; 1 from pulmonary embolism
2 weeks after the insertion of radium needles; 1, who was aged 70, appears to
have died from radiation sickness shortly after the termination of a course of
X-ray treatment, and 4 from radio-necrosis. There was also a patient who died
from late radio-necrosis of the rib 71 years after the operation, making 11 cases
in all.

Five-year Follow-up Results for All Cases.

Operation fatalities

Died under treatment

,, with cancer

without cancer .
Alive and well .

,,  with cancer

,, state unknown.
Untraoed .

Total died yearly

Percentage of all cases

First   Second   Third   Fourth   Fifth        Totals.
year.    year.   year.    year.   year.

34   .  -    .       .        .       .    34
5 .                         1. -.  -.  .

551   . 326   . 178    . 146   .   79     1280

10  .    11  .   13  .    9   .   19  .    62

..    .    ..  .   ..  .    .... 447]+22
..  .   ..   .   ..  .   .... 1097.       ..    664
... . ... .. ... .. ... .. 108)

.....~~~~~~4                  7             6 6   2 1 2 9

83              83
600   . 338   . 191    . 155   .   98   .  1382
. 282 . 15-9    . 90     . 7.3   . 4-6

The five-year survival rate for all cases was 31.2 per cent.

Of the 664 survivors, 47 are known to have died of cancer, and 13 without
cancer in the sixth and subsequent years.

Stage I

221

W. L. HARNETT

to0

1 C1

I   I   I    I

I       I   I

oo  r    o   eD

*    *   *

I  CX   I

,.   - ci

cia      P-
CO

qI> Cw  oo    C*  1-

14    to_ -           -

^=  I ,*  cq    e-  c   I es

I          I     CO   I
I .          .

*        *

?          .    ?    ?

Il I c

10  10    CO  10 , . . - 0

-   CO  o

o -

4 o

?~~~~

1Q, 1

.U: .~ ?

0

? . . ?

? o"O '"-S

0      m co

Cs   c,is  co"

c. 0  .+ ,-

222

4    m
.;4)

A) k

o 4

ul    eq     I  I r-

2               ,

iiO

-

oC

tHI I

* . . .

eq  I __r-

* . . .

if.

CO I X-
10.

*    .   .

c= r- es

Id.

'4

oi

'dCO

A 4)

00

-+a a

4C.

4) O
I -z

.4

cIt
t

N
t

P-14

i6

4-lA
9
w

.1

e

V

1?
0.

?i

-   cO

* . . .

- ICIO
* . ..

I*   .  , ..i ,- .

R* P- -F- 0
r- _- _4

CO
CO
1e

CO 0

P-

CO

.,

V

U,
ea

P4

m
0

0
.,.

0
0
"a

N
C)

O

D
m

o,-

N

r

. e

.$

* C;b

0

0

0

U)

ts

;or

o i

*s ts;

* CD * a2 .

0

co,

"a

0

0 G

; .

i. =

I,

%a

?4

z

STATISTICAL REPORT ON CANCER OF THE BREAST

I     I        A&       I

I                  I                   I                    [              I                    I

I  I ~~~~~  ~~  I                   -       ,c~~~~~~ ~~I

I  I  II        I.   I    I    I          II~~~~~~~~~~~~0

0     10     oa
-M -a

-   -   0

to~

I        I   I I  II

_ O~II
AC

I   I   I  I   I

s                    t
.0    _

10A          C11

CO     !             I         I   I     I   I         I     ?

.1     I    I     I    I     I

0     =    Ci     4         P-

t- 10   Co               04
04

.~ .,~ .~

-0

o       .    -.

,  g 0    Id.  -*

4,       ,      W

12   o

Eq  1il   H    E-

.? tlD,
0;

223

c1

c          -     14

CO
0

0

0

0
1-4
.

0114
04

$

0

0

w
0

t.
0

10

1..It

00

z

IC.00

at,

224

W. L. HARNETT

Analysis of Five-year Results by Methods of Treatment and Stages.

Surgical or Combined Methods.

Radical mastectomy  .

Known survivors     .
Per cent survived   .
Per cent of traced cases

Stage

I.

No. 236

. 144
. 61'0
. 68-2

Stage

II.
216

88
40.7
43.6

Radical mastectomy combined

with radiotherapy     . No.   69   . 136
Known survivors   .    .     .  41   .   49
Per cent survived  .   .     . 59.4 . 36.0
Per cent of traced oases  .    . 64.1 . 37.1
Local mastectomy         . . No.   61  .   19

Known survivors   .     .    .  34   .    4
Per cent survived  .   .     . 55.7 . 21.2
Per cent of traced cases  .    . 59.6 . 21.2

Stage
lila.

72
39
54.2
59.1

26
11
42.3
44.0

22

9
40. 9
40.9

Stage
IIIb.
163

41
25'2
25.6

105

31
29*5
30 1

21
4
19.0
19.0

Stage
IV.
15
0.0
*0'0

19
4
21.2
21.2

9
0.0
0 0

Not

staged.

1
0'0
0.0

38
16
. 42.1
. 42.1

1
1
.100.0
.100.0

Local mastectomy combined

with radiotherapy
Known survivors     .
Per cent survived   .

Per cent of traced cases

No. 26

16
. 61.5
. 64-0

Radium alone or with local surgery.

Interstitial radium alone  . No.    12

Known survivors    .    .    .    3
Per cent survived  .    .    . 25.0
Per cent of traced cases  .     . 30 0

23  .   12  .   24  .    8  .    7  . 100

8  .    6  .    4.     -   .    6  .   40
34.8 . 50.0 . 16-6 .     0.0 . 85.7 . 40.0
34.8 . 50)0 . 17.4 .     0.0 . 85.7 . 40)8

5
0(0
0.0

11   .  29   .   17  .   -

3   .    3.     -   .   -
27*3 . 10-3   .   0.0 .    -
27*3 . 10.3 .     0.0 .    -

74
9
12*2
12'5

Interstitial radium with surface

radium, teleradium  or X-
rays  .    .   .    . No.
Known survivors
Per cent survived

Surface radium or teleradium with!

without X-rays  .   . No.
Known survivors
Per cent survived

3  .    5 .

-   2   .

0.0 . 40.0 .

2  .  5  .  2   .   .  17

, --  . - .  . ~  .-  2

0.0  .  0.0  .  0.0  . .  11.8

-  .    2 .    9 .    8 .

-   .o  -  .   2 .   -.

-   . 0.0 . 22.2 . 00 .

11

1
9.9

Excision of tumour followed by

implantation of radium  No.  25
Known survivors  .    .    .   14
Per cent survived  .  .    . 56-0
Per cent of traced oases  .    . 636
Interstitial radium  followed by

local mastectomy .  . No.     1
Known survivors

Per cent survived  .  .    .  0.0

Teleradium followed by

radical mastectomy
Known survivors
Per cent survived

local or

. No.

10  .    4  .    3

6  .    2  .    3
60.0 . 50.0 . 100.0
66.6 . 50.0 . 100.0

1 .    2 .    3 .

2 .      1 .
0 0 . 100 0 . 33'3 .

.I -.         1 .

_-  .        1- ~ 1  .
0'0 .  -   . 1()'0 .

2 .    1 .   45

-.  1 .   26
0.0 .100.0 . 57.8
0.0 .100.0 . 63.4

-    -  ~.  7
-   .  -   .   3
-   .  -   . 42.8

-   .  -   .   2

1
-   .  -   . 500

Total.
703
312
44.4
47.6

393
152
38 7
39.9
133

52
39.1
40.6

-   .  30

3
-     . 10.0

STATISTICAL REPORT ON CANCER OF THE BREAST

X-rays alone or with radium and/or

local surgery.

H.V. X-rays alone   .    . No.

Known survivors   .    .
Per cent survived

Per cent of traced cases

H.V. X-rays followed by inter-

stitial radium  .    . No.
Known survivors .
Per cent survived

H.V. X-rays followed by inter-

stitial radium and local ex-
cision .   .      .    No.
Known survivors

Per cent survived  .   .    .

H.V. X-rays followed by local

mastectomy      .    .No.
Known survivors
Per cent survived

Local excision of tumour followed

by H.V. X-rays .     . No.
Known survivors        .
Per cent survived      .
Per cent of traced cases

Not treated by suraery or radiotherapy.

No.
Known survivors
Per cent survived

Stage

II.
22

3
13-6
13-6

Stage
lia.

25

3
12-0
12-0

Stage
IIIb.

89

5
5-6
5-7

Stage

IV.
114

2
1-8
1-8

10 .
0-0 .

7   .     5  .    11   .   26    .
3     .   1     .  4     .   7     .
42-8   . 20-0   . 36-4    . 26-9    .

--  2 .    2 .
--  0.0 . 0.0 .

4   .     5   .      7   .    15   .     3   .
.3    .     1   .     2   .     3   .      1   .
75-0   . 20-0     . 28-6    . 20-0    . 33-3    .

4
2
50.0
50.0

2
0.0
0-0

6  .   13  .   19  .
1 .      .     5.
16-6 .   0-0 . 26-3 .

1  .   --      6

-      .-   1

.- 16-6

-  34

10
. 29-4

1  .   21

-   11

0-0  . 45-8
0-0  . 47-8

2  . 232

8
0- 0 .  3-4

1

0-0
0-0

42 . 150 .

2 .-

4-9 . 0-0 .

Of the 8 surviving patients in this group, 2 died of cancer in the 6th and 8th
years respectively, 4 are still alive with ulcerating growths; the diagnosis of
malignancy has been changed in 1, and no details of the present condition of
the other are known.

Total results .

Known survivors
Per cent survived

Stage    Stage

I.      II.

. No.    485   .  467

.    274   .  170
. 56-5      . 36-4

Stage     Stage     Stage
IIIa.     IIlb.     IV.

224    .  538    .  362

85   .   102    .     8
37-9   .  19-0   .   2-2

Truscott (1947) gives the following percentage survival rates after five years
for all types of treatment of 836 cases, the cases being staged on the same principles
as in the above series.

Stage.

I
II

IIla
IIIb

Number.

250
484

24
78

Percentage

alive.
46-0
17-3
8-3
7-7

According to Table X in Truscott's paper there were 59-6 per cent known
5-year survivors of 114 followed-up stage I cases treated by surgery alone, and
25-8 per cent of 163 followed-up stage II cases. The corresponding figures in
the B.E.C.C. series are: 68-2 per cent of 210 followed-up stage I cases, and
43-6 per cent of 202 followed-up stage II cases.

16

Not

staged.

2
1
50 0
50.0

Total.
270

19
7-0
7-1

59
15
. 25.4

Not

staged.

53
25
47 2

Total.
2129

664
31 -2

225

W. L. HARNETT

Analysis of 5-year Results by Ages in Relation to Methods of Treatment.

Radical mastectomy

alone.

Total   Known      Per
number. survivors.  cent.

1      -        00
17        6     35-3
132       64     48 -5
221      106     48-0
210       88     41 -9
107       43     40 -2

14        5     35 7

702      312     44 4

X2= 4-29

P < 0-70 > 0-50

Radical mastectomy
with radiotherapy.

Total   Known      Per
number. survivors.  cent.

1
25
90
. 145

79
50

3
. 393

1
5
33
63
30
19

1

152

100.*0
20 -0
36 6
43.4
37 -.9
38 -0
33 -.3
38 -7

X2 =- 5-81

P < 0.50 > 0.30

Local mastectomy

alone.

Total  Known     Per

number. survivors.  cenlt.

2         2    100.0
2         1     50 0
8         5     62-5
22        9      40 9
27       10      37 0
46       16      34-8
26         9     34 -1

133       52     39 -1

x2 = 5-44

P<0.50>0-30

Similar calculations for the series of cases treated by local mastectomy com-
bined with radiotherapy, by radium with or without local surgery, and by X-rays
with or without local surgery showed, as the above figures do, that the variations
in survival rates of the different age-groups are not statistically significant.

Radical Mastectomy: 5-year Results by Duration of Symptoms.

The duration of symptoms at the time of operation is tabulated for 368
patients who had radical mastectomy, and for 177 who had radical mastectomy
with radiotherapy. The estimates of the duration of symptoms are based on
the patients' statements.

Duration of
Symptoms.

1 month and under .
1-3 months

3-6

Total under 6

months
6-12 months

Over 12 months

Total over 6

months

Duration not known
Differences in sur-

vival rate

Radical

mastectomy.

I      --l-

Number.   Known

survivors.

43       20
101       52
81       40

225      112 (50%)
56       19
77       29

133       48 (361 -1/o).

11        4

13 9 + 5-3.

Radical mastectomy
with radiotherapy.

A

Number. Known

N survivors.

16
46
44

106
27
41

4
20
20

Totals.

44 (41%)   .   331-156 = 47 -1%
11
20

68     31 (45 6%).      201-79 = 39-6%

3

- 41 +7-7.

7-5 ? 4 -4

These figures show that in the early cases radical mastectomy alone gives
the best survival rate, but that in cases of longer standing the survival rate was
improved by the use of radiotherapy in addition. The difference in percentage
is statistically significant for radical mastectomy, but not for combined treat-
ment.

226

Age

group.

15- .
25-

35- .
45- .
55- .
65- .
75- .

Totals

STATISTICAL REPORT ON CANCER OF THE BREAST

Analysis of 5-year Results by Site of the Growth in the Breast.

Radical mastectomy.

Stage I. 229.

Number.  Known     Per

survivors.  cent.
Upper and outer . 112       73     65 2

.. . inner .   56      34      60 - 7
Lower and outer.   35       20     57 1

. ... inner.      8        4     50.0
Central   .     .   18      11     61 1

Totals  .     . 229      142     62 -2

x2= 1.51

P <0.90>080

Stage II. 207.

Number.   Known     Per

survivors.  cent.
123       56      45 -5

24        6     25 -0
28       12     42-8

6        2     33-3
26       11     42-3
207       87     42 -0

X2 = 3-80

P < 0.50> (30

All stages. 668.

Known     Per
Number. survivors.  cent.

354     173      48 .9
106      48      45 -3
90      38      42 -2
31      15      48-3
87      37      42 -5
668      311     46-6

X2 = 2-34

P <070>0.50

It will be seen from the values of x2 and P that none of these variations
from the mean is statistically significant.

Radical mastectomy: effect on prognosis of slight attachment to the skin.

In the final staging on p. 219 it will be seen that cases in stages I and II with
slight attachment to the skin are separated from those in which there was no
attachment. This enabled the survival rate after radical mastectomy alone
and combined with radiotherapy to be worked out separately, with the following
result:

Stage I . Not adherent

Adherent

Stage II . Not adherent

Adherent .

Number.

128
177
122
230

Survived.

73
112
46
91

Per cent.

57 0
63.3
37.7
39*6

Difference.
} 63 ?i 57
} 1.9i5.4

These differences are not statistically significant.

Radical mastectomy: effect on prognosis of coincident pregnancy.

There were 10 patients who were pregnant at the time of discovery of the
growth in the breast, which was found to be in a relatively advanced stage in all
cases. Only 2 of them were treated by radical mastectomy, 1 of whom survived
5 years and 1 died of cancer in the 5th year.

Radical mastectomy: effect on prognosis of exploratory incision.

Data on this point were recorded for 1050 cases of radical mastectomy, with
or without radiotherapy in addition:

Known

Number. Survivors.

Per

cent.

Exploratory incision followed by mastectomy

immediately      ...

Exploratory incision followed by mastectomy

after an interval .   .    .

Mastectomy without exploratory incision .
Not stated   .     .    .    .     .

95   .    58   .   61.1

65
536
354

38
210
138

58.5
39.3
39'0

227

W. L. HARNETT

The differences in survival rate between those in whom an exploratory incision
was made (160), and those in whom this was not done (536), are statistically
significant and are probably due to the fact that the former were early cases in
which the diagnosis was in doubt. The differences between the two groups in
which an exploratory incision was made are not statistically significant.
Ulceration of skin: effect on prognosis (all treatments combined).

The survival rate of cases with fixation to the skin and with actual ulceration
were worked out separately. The figures are:

No lymph nodes involved:    Number.    Survived.   Per cent.     Difference.

Growth fixed to skin   .     41   .     22    .   53 7
Skin ulcerated    .    .     73   .     21    .   288

Lymph nodes involved:

Growth fixed to skin   .    104    .    24    .   23-1          4   4 8
Skin ulcerated    .    .    204   .     32    .    157

Ulceration has a serious effect on prognosis, but the difference in the survival
rate is only statistically significant in those cases where no lymph nodes are
involved.

Estimation of Survival after Treatment.

Dr. Stocks, to whom this question was referred, advised that unless the
follow-up of cases makes it possible to assign accurately every death either to
cancer on the one hand or to intercurrent causes on the other, the only sound
method of dealing with the duration of survival is an actuarial one, which means
calculating from a life-table the total months which would be lived in the period
of observation by a group of people in the general population having the same
sex-age distribution as the group of patients dealt with. This gives the mean
number of months expected to be lived during the five years by each group.
The mean number of months actually lived is then calculated and expressed as a
percentage of the normal expected for that group, making allowances for cases
followed up for less than five years.

English Life Table No. 10 (1930-32) was used for ascertaining the expectation
of life.

Stage       Stage     Stage      Stage
I?adical mastectomy alone.               I.         II.    IIIa.     IIIb.

Number of cases of known duration  .  .  210  .   198  .      68  .    158

rMaximum

Mean number of months lived   possible  60-00 .  60-00 .    59-12 .   59-77

in 5 years from onset  .  Expected  .   5720 .  57 16 .   56 -02 .  5704

LActual   .    5201 .    43-2 4372859 .       39-02
Per cent of Expected  .  91-03 .  75-72  .  84-95 .   68-41

Radical mastectomy with post-operative radiation.

Number of cases of known duration .  .  55 .      116  .     21  .      78

Maximum

Mean number of months lived J  possible  60 00 .  60-00 .   59.43 .   60-00

in 5 yearsfromonset  .  Expected .   57.95 .   57-89 .      56-78  .  57-36

FActual  .     51-16 .   40.53 .    47-81 .   41-73
Per cent of Expected .  88-28 .  70-01 .    84-20 .   72-75

228

STATISTICAL REPORT ON CANCER OF THE BREAST

Stage       Stage       Stage       Stage
Radical mastectomy with pre-operative radiaticn.  I.       II.        IIIa.        IIIb.

Number of cases of known duration   .          3    .      7    .      3    .     18

Maxim um

Mean number of months lived       possible  60-00   .   60-00   .   60-00   .   60-00

in 5 years from onset   . Expected     .  57-63   .   58-57   .   57-63   .   57-63

LActual      .   46-66   .   32 -00  .   53-66   .   37.77
Per cent of Expected  .   80-93   .   54-64   .   93 11   .   65-54
Radical mastectomy with implantation of radium.

Number of cases of known duration   .    .     3     .     6     .     2          16

FMaximum

Mean number of months lived       possible  60 -00   .  60-00   .   60-00   .   60-00

in 5 years from onset   . Expected     .  59 -16   .  58 -02  .   48 04   .   56 -43

LActual      .   60-00   .   38 -00  .   35-50   .  45-83
Per cent of Expected  . 101-42    .   65-49   .   73 -90  .   81 -22
Local mnwetectomy alone.

Number of cases of known duration   .    .    56     .    18     .    21     .    20

Maximum

Mean number of months lived       possible  60-00    .  60-00    .  60-00    .  60-00

in 5 years from onset   . Expected     .  53-08    .  52-88    .  53-27    .  53 -07

LActual      .   51-82   .   40-28   .   46-38   .   37-15
Per cent of Expected  .   97-63   .   76-17   .   87 -07  .   70 -00

Local mcastectomy with post-operative radiation.

Number of cases of known duration       .     23    .     22    .     12          23

'Maximum

Mean number of months liveti      possible  60-00    .  60-00    .  60-00    .  60-00

in 5 years from onset   . Expected     .  54-64    .  56-49    .  54-31    .  55 -09

.Actual      .   55 -22  .   42 -09  .   49 33   .   39 39
Per cent of Expected   . 101-06   .   74-51   .   90-83   .   71 -50

Interstitial radium alone or with surface radium.

Number of cases of known duration   .    .    13     .    10     .    12     .    32

Maximum

.Mean number of months lived       possible  59-08   .   60-00   .   57 .00  .   60-00

in 5 years from onset   . Expected     .   52-56   .   55 -53  .   50-59   .   55-42

Actual      .   41-38   .   32-80    .  37-83    .  29-16
Per cent of Expected   .  78-73    .  59-07    .  74-78    .  52-62
Interstitial radium with local surgery.

Number of cases of known duration   .    .     23     .    10     .     6     .    6

(Maximum

Mean number of months lived        possible  60-00   .   60-00   .  60-00    .  60-00

in 5 years from onset   . Expected     .   57-12   .  58-54    .  58-16    .  55-96

Actual       .  49-91    .  47-20    .  55-17    .  47-50
Per cent of Expected   .  87 -38   .  80-63    .  94-86    .  84-88
X-rays alone or with interstitial radium.

Number of cases of known duration   .    .    25     .        2  ,    34         112

Maximum

Mean number of months lived J      possible  60 -00  .  6000     .  60 00    .  60 00

in 5 years from onset   . Expected     .  52-08    .  52 -18   .  53-55    .  54-87

LActual       .  41-48    .  33 -40   .  37 -38   .  34 -12
Per cent of Expected   .  79-65    .  64-00    .  69-80    .  62-18
X-rays with local surgery.

Number of cases of known duration   . ,       21           9           9     ,    19

Maximum

Mean number of months lived        possible  58-86   .60-00         60-00       6 0 00

in 5 years from onset   . Expected        55-38       55-61       53-77       54-80

Actual       .  49 -52   .  48 -33   .  39 -33   .  42-00
Per cent of Expected   .  89-42    .  86-91    .  73-14    .  76-64

229

W. L. HIARNETT

The general conclusions to be drawn from the figures given above are:

1. The 5-year expectation of life after radical mastectomy ranges from
91.03 per cent of normal in stage I down to 68-4 per cent of normal in
stage IlIb.

2. The 5-year expectation of life is not increased by post-operative
radiation in stages I, 11 and IIia, but in stage IIIb (78 cases) there was
an increase of 4.3 per cent.

3. The number of patients treated by radical mastectomy with pre-
operative radiation or implantation of radium at the time of operation
was too small to admit of reliable conclusions being drawn from the
figures.

4. Local mastectomy was the method of treatment in 17.0 per cent of
those treated by operation, and, though the actual period of survival was
much the same as after radical mastectomy, the 5-year expectation of
life was relatively higher on account of the high mean age of these patients.
These results were slightly improved by post-operative radiation.

5. Local excision of the tumour followed by interstitial radium or
X-rays was the method used in a few cases, and gave a 5-year expectation
of life almost equal to that afforded by radical mastectomy in stage I,
and better than the latter in patients who were in the later stages,
especially in stage IIIb, but the numbers so treated were small.

6. Radiotherapy alone gave a lower expectation of life.

7. It was found that patients in stage IV who were not treated either
by surgery or radiotherapy had a 5-year expectation of life of 46-7 per
cent of normal, which was not improved by palliative X-ray treatment.

All these figures must be considered in the light of the fact that the mean
duration of the disease is 38.3 months (Major Greenwood, 1926), so that these
patients whose mean age was 57 years would have a 5-year expectation of life
of about 56 months; a 5-year follow-up is therefore insufficient for assessment
of the results of treatment. The variations in mean age of the patients sub-
mitted to various types of treatment is shown below, and indicates that the
more radical methods were used in the younger patients.

Years.

Mean age of 703 patients treated by radical mastectomy alone  .  .  54. 11

393   ,,       ,,      ,,        ,,    combined with

radiotherapy    .    .    .    .    .    .    .    .    .    .   51 18
Mean age of 133 patients treated by local mastectomy alone  .  .   63 44

,,  ,,  100   ,,      ,,      ,,      ,,    combined with

radiotherapy    .    .    .    .    .    .    .    .    .    .   59*50
Mean age of 174 patients treated by radium alone or with local surgery  58 25

,,  ,, 393    ,,      ,,    H.V. X-rays alone or with radium

and/or local surgery  .   .    .    .    .    .    .    .    .   60 46
Mean age of 232 patients not treated by surgery or radiotherapy .  .  64-16

230

STATISTICAL REPORT ON CANCER OF THE BREAST               231

Pathological Report.

Number.   Per cent.

No histological examination before or after death  .  .  743   .  35 0
Histological examination done before or after death .  .  1386  .  65.0

Result doubtful   .    .    .    .    .    .    .      1
The specimen examined was non-malignant    .    .      7
Carcinoma, type unspecified  .   .    .    .    .    106
Spheroidal cell carcinoma   .    .    .    .    .    980
Adenocarcinomna   .    .    .    .    .    .    .    190

,,  colloid  .   .    .    .    .    .    28
,,  papillary    .    .    .    .    .     6
Squamous cell carcinoma, keratinizing  .   .    .      2

non-keratinizing   .    .     2
Sarcoma (type not specified 3, spindle cell 2, fibro-

sarcoma 2, reticulum cell 1) .  .   .    .    .      8
Paget's nipple    .    .    .    .    .    .    .      3

,,  ,,  with spheroidal cell carcinoma  .  .     7
,,  ,,  ,, adenocarcinoma    .      .    .      1
Duct carcinoma    .    .    .    .    .    .          45

Histological Grade (according to Broders' classification).

Figures for this are omitted owing to the small proportion of cases graded.

Histological report on lymph nodes.                            umr.

Number.

No nodes found involved on histological examination .  .  359
Axillary nodes     ,,         ,,          ,,     .    .   625
Apical axillary (subclavicular) nodes found involved .  .  36
Nodes not examined histologically  .  .     .    .    .   402

The percentage of cases in which there is disagreement between the clinical
and histological findings is shown by the following figures:

984 cases in which a histological examination was made.

Number.   Per cent.

No nodes involved clinically; confirmed on histological

examination     .    .    .    .    .    .    .     .  252   .  61 3
No nodes involved clinically, but found involved on histo-

logical examination  .    .    .    .    .    .     .  159   .  38 7

411      100.0
Nodes involved clinically, confirmed on histological examina-

tion  .    .    .    .    .    .    .    .    .     .  466   .  81  3
Nodes involved clinically, but found not involved on histo-

logical examination  .    .    .    .    .     .    .  107   .   18 7

?*~~~                                        ~~~~ ~~~573  100.0

W. L. HARNETT

38.7 per cent more patients had the nodes involved than was evident clinically,
whilst in those with nodes clinically involved the enlargement proved not to be
malignant in 18.7 per cent.

Basis of diagnosis in 1018 cases.

Clinically malignant:                               Number.  Per cent.
Confirmed by histological examination and/or autopsy  .  677  .  66 5

,,  appearance of metastases or recurrence  .  201   .  19 7
Diagnosis based on clinical evidence only .  .  .    .  123   .  12- 1

Clinically benign:

Proved malignant by histological examination  .  .   .   15   .   1.5

~,,  ,,      appearance of metastases or recurrence  2  .  0 2

Other Primary Growths.

There were 60 patients who had growths in both breasts. In 54 patients the
growth in the opposite breast was obviously a metastasis from the primary
growth, but there were 6 patients in whose cases the second growth appeared to
have started almost simultaneously in both breasts. There were also 23 patients
who had had the opposite breast amputated for carcinoma from 14 to 30 years
previously. The average interval between the two growths was less than 5
years in 9 cases, 5-10 years in 8 cases, over 10 years in 5 cases, and unknown
in 1. The mean of the intervals was 8.0 years.

Five patients had had cancer of some other organ for which they had been
successfully treated; 11 had simultaneous growths in other organs-bladder 1,
uterus 2, lung 2, rodent ulcer 2, stomach 1, vulva 1, rectum 1, ovary 1; and
there were 4 patients in whom second primaries appeared after the breast growths
had been removed-rectum 2, cervix 1, rodent ulcer 1. None of these patients
survived.

Cause of Death in 1382 Patients.

Number.

Cachexia  .    .     .    .    .    .    . 1042
Cardio-vascular disease   .    .    .    .   47
Cerebral metastases  .    .    .    .    .   21
IJraemia  .    .     .    .    .    .    .    6
Pulmonary complications  .     .    .    .   95
Sepsis    .    .    .     .    .    .    .    14
Surgica] shock  .   .    .     .    .    .    7
Haemorrhage    .    .     .    .    .    .    3
Pulmonary embolism  .    .     .    .    .    16
Intercurrent disease or unknown cause .  .   131

Fourteen of the sixteen deaths from pulmonary embolism were post-operative
at a mean interval of 11-5 days; in 1 case the operation had been performed
18 months previously and in 1 case there had been no operation. The diagnosis

232

STATISTICAL REPORT ON CANCER OF THE BREAST

was confirmed by autopsy in 12 cases, in 4 of which thrombosis of the femoral,
subclavian or saphena vein was found to be the source of the embolus.

No autopsy .
Autopsy done

Autopsy.

Number.
.      .    .    1246
.        .    ..    136

Relevant autopsy findings (multiple in most cases).

Local growth only  .   .   .    .    .
Extension to neighbouring parts

Metastases in supraclavicular lymph nodes.

,,  mediastinal lymph nodes

,,  lungs, pleurae and thoracic organs.
,,  liver and abdominal organs
,,  brain  .  .    .
,,  skeletal system  .
Pulmonary complications .  .
Abdominal      ,,      .   .

No growth found (post-operative deaths)

Per cent.
*     90-1

9.9

Number.
.   22

.  20

22
.  24

55
62
*      .  11

32
41

7
11

RECURRENT CASES-FEMALES.

There were 374 recurrent cases. The type of recurrence, nature of treatment
of the primary, and the intervals from operation to first recurrence are shown in
the following table.

Treatment of .

primary.

Surgery alone

Surgery combined wit

radiation

?  ~~~~~~~~~Mean

Local      Metas-    Local and  interval in
Number.                         ~~~~~~~~~~~interval in

recurrence.  tases.   metastases.

months.

45          . -  .    - .       28.1
~~~~~~~-                           . 292
292-              .   169    .    -     .   37 9

-  .  --    .    78    .   33- 1

h

63  (
63t

9

30

24

Radiotherapy alone

4
19       _

I _

? ?

6  .  -

9

-- *  9 v

36-6
23.7
30-6

10-3
34.6
37.4

Totals .

374

58   .   205

111

The longest intervals were in 2 patients who were found to have metastases
in the lung (confirmed histologicaIly) 23 and 20 years respectively after radical
mastectomy. There were also 2 patients who had local recurrence (not confirmed

233

W. L. HARNETT

histologically) 16 and 17 years respectively after radical mastectomy. It will be
seen that metastases were four times as frequent as local recurrence after radical
surgery, and occurred most commonly in the fourth year after operation. The
number of patients who had had post-operative radiotherapy was too small
for the differences in times of appearance of local recurrence and metastases to be
statistically significant.

The patients with local recurrence had a 5-year survival rate of 12 1 per cent.
In the other two groups palliative treatment was given in less than half the
patients, and the 5-year survival rates were 3.9 and 2-7 per cent respectively.

PRIMARY CASES-MALES (23).

Age distribution.

Age distribution.

45-49
50-54
55-59
60-64

Number.

2
1
4
7

Age distribution.

65-69
70-74
75-79
80-

Mean age  .

Standard deviation
Difference of means

Males.

63-5 i 1* 7
84 + 1-2

6 5 +

Females.

570 ? 0.4
12.6 ? 0-2
1.78

The difference between the mean
significant.

ages of males and females is statistically

Family history of cancer.

Fifteen patients gave no family history of cancer, and the question was not
answered for the remaining 8.

First symptom.

Lump in breast   .    .    .    .
Pain in breast                  .

" Eczema" or ulceration of skin of breast
Bloody discharge from nipple .  .

No symptoms-tumour discovered during routine

examination    .    .    .    ..

Number.   Per cent.

15   .   65*2

1   .    4.3
5   .   21*7
1   .    4.3
1   .    4.3

Interval from first symptom to first consultation.

34.8 per cent of the patients consulted a doctor within 3 months of noticing
the first symptom, 26-1 per cent between the third and sixth months, and in
39.1 per cent more than 6 months had elapsed.

Number.

5
1
2
1

234

STATISTICAL REPORT ON CANCER OF THE BREAST

Findings on examination.

Location of tumour:

Right breast .    .    .
Left    ,,   .    .    .
Upper and outer quadrant
Central, behind nipple  .
Lower and outer quadrant

Diffuse, too advanced to identify primary site

Size of tumour:

Small-up to 4 sq. cm.
Medium-4-20 sq. cm.
Large-20-100 sq. cm.

Number.     Per cent.

12    .   52- 2
11    .   47-8
5    .   21-7
15    .   65-2

2    .     8.7
1    .    4.3

5
11

7

21-7
47'8
30-4

Clinical groups.

Stage I:

Confined to breast; lymph nodes not involved clinically

~~~~),,  ,,  ,,         ,,     histologically .

Stage 1I:

Axillary lymph nodes involved clinically .

,,     ,,         ,,   histologically

Stage IIIa:

Fixed to skin or pectoral fascia, no nodes clinically

Stage IlIb:

Fixed to skin or pectoral fascia, with nodes clinically

,,      ,,        ,,           ,,     histologically

Stage IV:

Supraclavicular lymph nodes involved
Remote metastases present  .

The percentage of patients who were still in Stage I was only 8-7.

Number.

1
1
2

4
. .  2

6

4

3
4
11

2
.  .     2

4

235

W. L. HARNETT

Methods of treatment and 5-year results.

OpNumber

Number. fata

Radical mastectomy    .    8
Radical mastectomy fol-

lowed by H.V. X.rays .   2
Local mastectomy .    .    3
Local mastectomy followed

by H.V. X-rays .    .    3
H.V. X-rays alone .   .    1
H.V. X-rays followed by

interstitial radium  .   2
Not treated  .   .    .    4

Totals .

23

ration Survived
lities.   5 years.

1     .     2

Died
with

cancer.

1

Died

without
cancer.

3

Not

traced.

1

Operation
fatalities.
Per cent.

12 -5

-       .      I    .      1     .           .I

I-           .      2     .    -       .    -

2     .    -       .      1    .      -

?-         .o           .      1     .            .           .       _-I

-  .   2
-  .   3

1

1     .      4     .     12      .      3     .      3

Five-year follow-up results for all cases.

First
year.

Operation fatalities
Died with cancer .

,, without cancer .
Alive and well .

,, with cancer

Untraced .       .

Total died yearly .

1
8

9

Second
year.

2
2

*  .  o

Third     Fourth
year.      year.

2    .   -

4

The 5-year survival rate for all cases was 17-4 per cent.

Five-year survival rate according to stages.

Stage.                  Number.

I

II

IIIa

Illa

IIIb
IV

2
6
4
8
3

Known

survivors.

2
1
1

The numbers in each group are so small that a detailed analysis of survival
rate by methods of treatment and stages would not be statistically significant.
An actuarial estimation of 5-year expectation of life of 8 patients who had radical
mastectomy (Stage I, 2; Stage II, 2; Stage IIIa, 1; Stage IIIb, 3) gave an
actual mean survival period of 36 months, which was 72-48 per cent of normal.
If 2 patients who had post-operative radiotherapy are included, the figures are
38-9 months. and 75-31 per cent of normal expectation.

Pathological report.

No histological examination before or after death

Histological examination done before or after death

Spheroidal cell carcinoma   .    .
Adenocarcinoma      ...
Duct carcinoma .      .     ..

Basal cell carcinoma          . .

Number.     Per cent.

8
15
10

1
2
2

34-8
65-2

236

Fifth
year.

1
3
1
3

Totals
1

12  16

31

4  23
3

Per cent.

33-3
25-0
12-5

STATISTICAL REPORT ON CANCER OF THE BREAST

Histological report on lymph nodes.

No nodes found involved on histological examination .  2
Axillary nodes     ,,         ,,         ,,      .    9
Nodes not examined histologically  .   .    .    .    4

In 2 of the 9 positive cases the nodes were not involved clinically.

Basis of diagnosis.

Number.   Per cent.

Clinically malignant, confirmed by histological examina-

tion  .    .    .    .    .    .    .    .    .   15       65   2
Clinically malignant, confirmed by appearance of

metastases or recurrence  .    .    .    .    .    4   .   174
Diagnosis based on clinical evidence only  .  .  .    4   .   17.4

Cause of death in 16 patients.                          Number.

Cachexia   .    .     .    .    .    .    .    8
Cardio-vascular disease .  .    .    .    .   2
Pulmonary complications    .    .    .    .   4

embolism   .    .    .    .    .    1
Unknown    .    .     .    .    .    .    .    1

Autopsy.

Autopsy was performed on 1 of the 16 patients who died, and multiple
metastases were found.

RECURRENT CASES-MALES.

There were 3 patients with recurrences following radical mastectomy in 2
cases, and local mastectomy in 1. All 3 had had post-operative radiotherapy,
and were in the third year following operation. All had metastases and died
within a few months.

SUMMARY.

1. A statistical analysis of 2529 cases of cancer of the breast. 2152 of these
were primary cases, 2129 females and 23 males.

2. 22.1 per cent of the female patients were single women and 77.8 per cent
were married or widowed. The mean age of the single women was 55.6 years
and that of the married and widowed 57.4 years.

3. The percentage of childless marriages amongst 1658 married and widowed
women was 16-1 as compared with 12-5 in published control series.

4. A lump in the breast was the first complaint in 77.4 per cent of the patients,
pain either local or referred in 11-6 per cent, discharge from the nipple in 2-2 per
cent, and symptoms due to metastases in 1.6 per cent. In 1.6 per cent the tumour
was discovered by the doctor or during routine examination, having caused no
symptoms.

237

W. L. HARNETT

5. 44-1 per cent of the patients consulted a doctor within 3 months of noticing
the first symptom, 15.3 per cent during the next 3 months, and in 33.3 per cent
the symptoms were of over 6 months' duration before advice was sought.

6. 85-6 per cent of those who consulted a doctor were referred to hospital at
once, but 3-6 per cent were told that the condition was not serious, and 4.8 per
cent were treated by palliative methods for more than 6 months.

7. On admission to hospital it was found that 59.7 per cent of the patients
had clinical involvement of the axillary lymph nodes and 10-3 per cent had
clinically recognizable distant metastases. Four stages were defined, and in
the final grouping 22.8 per cent of the patients were placed in Stage I, 21.9 per
cent in Stage II, 35-7 per cent in Stage III and 17'1 per cent in Stage IV.

8. 703 patients were treated by radical mastectomy alone with an average
5-year survival rate of 47.6 per cent of traced cases, ranging from 68.2 per cent of
those in Stage I down to 25.6 per cent in Stage IIIb; the operation mortality
was 3.1 per cent.

393 patients were treated by radical mastectomy combined with radio-
therapy, with an average 5-year survival rate of 39.9 per cent of traced cases,
ranging from 64.1 per cent of those in Stage I, down to 30.1 per cent of those in
Stage IIIb. Local mastectomy alone in 133 patients gave a 5-year survival rate
of 59*6 per cent of traced cases in Stage I, down to 19.0 per cent in Stage IIIb;
these results were improved when local mastectomy was supplemented by radio-
therapy in 100 cases. The patients treated by local mastectomy were on the
average older than those treated by radical mastectomy.

9. Treatment by radium and X-rays was usually only palliative, but there
was a group of 45 cases in whom local excision of the tumour was followed by
implantation of radium, with a survival rate of 63.4 per cent of traced cases for
all stages.

10. The results of different methods of treatment were assessed by actuarial
methods, in which allowance is made for the ages and consequent expectation of
life of the patients. It was found that radical mastectomy gave a 5-year expecta-
tion of life ranging from 91.03 per cent of normal in Stage I, down to 68.4 per
cent of normal in Stage IIIb; post-operative radiation improved these results
by 4-3 per cent in Stage IIIb, in other stages the results were not so good as those
of radical mastectomy alone. Other methods of treatment were assessed in
the same way.

11. 31.2 per cent of all patients survived 5 years, ranging from 56.5 per cent
of those in Stage I, down to 19.0 per cent of those in Stage IIb, and 2.2 per
cent of those in Stage IV.

12. Histological examinations were made in 65.0 per cent of the patients;
38.7 per cent who had no clinical evidence of involvement of the axillary lymph
nodes were found on microscopical examination to have them involved; on the
other hand, nodes which were considered on clinical grounds to be malignant
were found in 18.7 per cent not to be so on microscopical examination.

13. There were 20 male patients, whose mean age was 63.5 years. Their
5-year survival rate was 17.4 per cent.

The Committee wish to thank Mr. A. J. Durden Smith, Dr. Frank Ellis and
Mr. Stanley Lee, who constituted the Sub-committee which revised and edited
this report, for their valuable advice and help.

238

SPREAD OF PYLORIC CARCINOMA                239

REFERENCES.

BUSK, T., AND CLEMMESEN, J.-(1947) Brit. J. Cancer, 1, 345.

EWING, J.-(1940) 'Neoplastic Diseases,' 4th ed. Philadelphia and London (Saunders

Co.).

GREENWOOD, M.-(1926) Ministry of Health Reports, 33.
HARRINGTON, S. W.-(1938) Minn. Med., 21, 1-8.

LANE-CLAYPON, J. E.-(1926) Ministry of Health Reports, 32.

REGISTRAR-GENERAL.-(1938) Decennial Supplement England and Wales, 1931,

Pt. IIa, Occupational Mortality, 149.

STOCKS, P., AND KARN, M. N.-(1933) Ann. Eugen., Camb., 5, 237.
TRUSCOTT, B. M. N.-(1947) Brit. J. Cancer, 1, 129.

WAINWRIGHT, J. M.-(1931) Amer. J. Cancer, 15, 2610.